# The persistent DDT footprint of ocean disposal, and ecological controls on bioaccumulation in fishes

**DOI:** 10.1073/pnas.2401500121

**Published:** 2024-10-28

**Authors:** Lillian McGill, Toni Sleugh, Colleen Petrik, Kenneth Schiff, Karen McLaughlin, Lihini Aluwihare, Brice Semmens

**Affiliations:** ^a^Marine Biology Research Division, Scripps Institution of Oceanography, University of California, San Diego, La Jolla, CA 92093; ^b^Integrative Oceanography Division, Scripps Institution of Oceanography, University of California, San Diego, La Jolla, CA 92093; ^c^Southern California Coastal Water Research Project, Costa Mesa, CA 92626; ^d^Geosciences Research Division, Scripps Institution of Oceanography, University of California, San Diego, La Jolla, CA 92093

**Keywords:** DDT, Fish, sediment, marine, spatiotemporal modeling

## Abstract

Globally, ocean dumping of chemical waste is a common method of disposal and relies on the assumption that dilution, diffusion, and dispersion at ocean scales will mitigate human exposure and ecosystem impacts. In southern California, extensive dumping of agrochemical waste, particularly chlorinated hydrocarbon contaminants such as DDT, via sewage outfalls and permitted offshore barging occurred for most of the last century. This study compiled a database of existing sediment and fish DDT measurements to examine how this unique legacy of regional ocean disposal translates into the contemporary contamination of the coastal ocean. We used spatiotemporal modeling to derive continuous estimates of sediment DDT contamination and show that the spatial signature of disposal (i.e., high loadings near historic dumping sites) is highly conserved in sediments. Moreover, we demonstrate that the proximity of fish to areas of high sediment loadings explained over half of the variation in fish DDT concentrations. The relationship between sediment and fish contamination was mediated by ecological predictors (e.g., species, trophic ecology, habitat use), and the relative influence of each predictor was context-dependent, with habitat exhibiting greater importance in heavily contaminated areas. Thus, despite more than half a century since the cessation of industrial dumping in the region, local ecosystem contamination continues to mirror the spatial legacy of dumping, suggesting that sediment can serve as a robust predictor of fish contamination, and general ecological characteristics offer a predictive framework for unmeasured species or locations.

## Significance

Extensive ocean disposal of the pesticide DDT occurred for most of the last century in Southern California. Our study compiles a large database of DDT measurements for fish and marine sediments, showing that despite over 50 y since the cessation of industrial dumping, the spatial footprint of DDT disposal is well conserved in sediments. We find evidence for context-dependent controls on DDT bioaccumulation in fish and illustrate how major drivers of bioaccumulation shift across a gradient of sediment contamination. Findings provide a generalizable framework for predicting DDT burdens in fish using location and ecology and support a cautionary approach to future ocean dumping of chemicals, where place-base impacts dominate the prediction of contaminant burdens in fisheries for decades.

Concerns about synthetic chemicals gave rise to the modern environmental movement in the early 1960s (1), yet the transport, storage, and fate of these contaminants within ecological communities remain poorly understood (2, 3). For example, legacy chlorinated hydrocarbon contaminants, including organochlorine pesticides such as DDT, continue to pose health risks to humans and wildlife despite a ban on their use in the United States half a century prior (4–6). Globally, DDT is still used in some parts of the world for vector control (7). The persistence of DDT and its breakdown products, cumulatively referred to as DDX, in the environment is due to its resistance to degradation (8). Similar to other organic contaminants, low water solubility, high partitioning to organic matter and lipids, and resistance to biodegradation result in the accumulation of DDX in sediments, bioaccumulation in organisms, and biomagnification through food webs (9–11). The presence of organic contaminants like DDX in fish tissues poses human and ecosystem health risks, and resource managers would benefit from tools to characterize relative risks in undersampled regions or species.

Bioaccumulation mechanisms of DDX by marine fishes are well understood and reflect a combination of uptake and elimination processes. Generally, the concentration of contaminants in an organism’s diet, associated with habitat or trophic position over appropriate temporal and spatial scales, represents the primary route of exposure to higher trophic level consumers such as fish (12–14). Individual or species-level differences in growth efficiency, reproductive offloading, and the partitioning capacity of the organism for the contaminant, which is primarily determined by the whole-body lipid content of the organisms, can also be important (15–19). Although individually the factors that impact bioaccumulation are well understood, there is limited knowledge of how complex, cooccurring interactions affect contaminant uptake across species and habitats (20). Particularly lacking is an understanding of how spatial variability in sediment contaminants interact with ecological processes to drive overall bioaccumulation. Understanding these factors, and the relative importance of their roles in bioaccumulation, is essential for predicting where risks of exposure may be high for humans and wildlife.

**Fig. 1.**
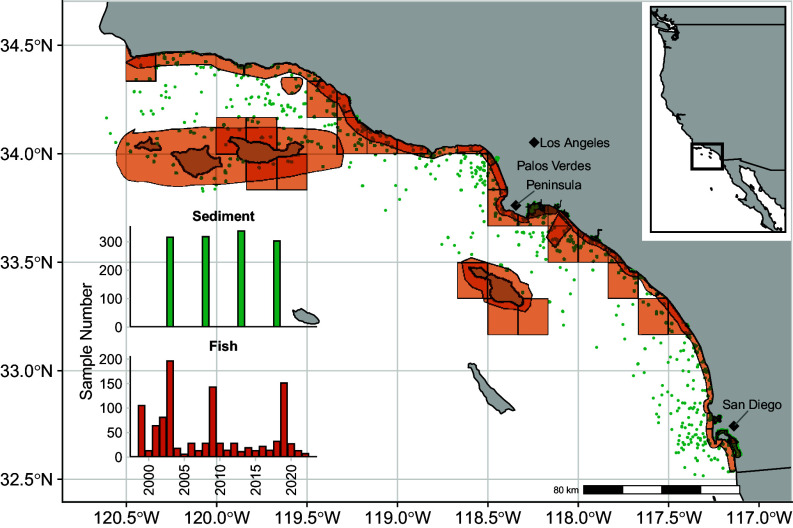
Map of sediment sampling locations and fishing zones, and the number of sediment and fish samples in our dataset through time (*Inset*). Nearshore polygons are derived from McLaughlin et al. (32) and blocks are California Department of Fish and Game 256 km^2^ fishing blocks.

In heavily industrialized or agricultural regions, such as coastal areas of the Southern California Bight (SCB), concentrations of contaminants can be particularly high. For example, DDX concentrations up to 200 μg g^−1^ dry weight (dw) have been reported in SCB sediments (21). Repositories of contaminants stored in marine sediments can be remobilized through bioturbation and resuspension of sediments, acting as chronic sources of these compounds (22–25). Within the SCB from 1947 to 1971, North America’s largest producer of the pesticide DDT, Montrose Chemical Corporation, discharged its industrial waste both through the Los Angeles County wastewater treatment plant, which was deposited nearshore on the Palos Verdes Shelf (PVS), located directly off the Palos Verdes peninsula (Fig. 1), and via ships that transported and dumped bulk waste in deeper waters (26–28). The PVS was designated as a Superfund Site by the US Environmental Protection Agency in 1996, and resultingly, extensive monitoring of contaminants in fish tissue and sediments has occurred (29). However, the offshore dumpsites were largely overlooked until recently, when visual confirmation of offshore disposal was provided by Remotely Operated Vehicle footage (27). This has ignited an urgency to understand the extent and impact of the waste that was released within the region (30) (*SI Appendix*, Fig. S1). For DDX, this legacy of pollution has resulted in a gradient of sediment contamination spanning several orders of magnitude across the SCB (31).

The SCB presents a unique opportunity to study fish bioaccumulation due to the large and complex gradients of DDX contamination across 78,000 km^2^ of coastal ocean and the legacy of economically and culturally valuable fisheries in the region (33). Due to its long history of anthropogenic impacts, contaminant monitoring efforts for sediment and fish in the SCB have been ongoing for more than two decades, resulting in a wealth of available data (34). We compiled a spatially explicit database of existing sediment and fish DDX measurements to examine how this legacy of regional ocean dumping translates into the contemporary contamination of the coastal ocean. Through a synthesis and analysis of these data, we aim to answer the following questions:

1. How is DDX distributed in sediments throughout the SCB?
2. What is the relationship between fish and sediment DDX concentrations, and how do ecological factors mediate this relationship?

To date, contaminant-related health advisories for seafood consumption have generally focused on taxonomy alone to determine which species are safe to eat and which bear risk. By answering the questions above, we aim to lay the groundwork for predictive models of DDX exposure via seafood consumption that are spatially explicit but generalizable across taxa with shared ecologies. Moreover, our findings will link the long legacy of regional ocean dumping to contemporary ocean ecosystem processes and fisheries of southern California.

## 1. Results

We obtained spatially resolved sediment and fish data from nine primary sources collected between 1998 and 2021 (Fig. 1 and *SI Appendix*, Table S1) (35). Of the 45+ DDX congeners that have been identified within the SCB (6), we examined the six most commonly monitored and report all DDX concentrations as the sum of 2,4′-dichlorodiphenyldichloroethylene (DDE), 4,4′-DDE, 2,4′-dichlorodiphenyldichloroethane (DDD), 4,4′-DDD, 2,4′-DDT, and 4,4′-DDT. For samples with detectable DDX concentrations, DDE was the primary compound present in both sediment and fish samples (*SI Appendix*, *Supporting Text* and Fig. S2). Sediment DDX concentrations, [DDX_sed_], were reported in ng g^−1^ dw and fish tissue DDX concentrations, [DDX_fish_], were lipid-normalized by percent lipid prior to analyses and reported in ng 0.01 g^−1^ lipid. We chose to lipid normalize fish concentrations due to the well-documented, positive relationship between lipid content and organic contaminant concentration in fish tissue (16). We assigned each fish species represented in our dataset diet and habitat classifications according to their adult life history characteristics (*SI Appendix*, Tables S2 and S3). Diet categories included herbivores, primary carnivores, secondary carnivores, and tertiary carnivores, and habitat categories included pelagic, midwater, and demersal with the demersal group subdivided again into benthic (species that rest on the ocean floor) and benthopelagic (species found in the water just above the ocean floor). Our final dataset consisted of 1,275 sediment samples and 1,074 fish tissue composites from 61 species. Our primary statistical framework consisted of two components. First, we fit spatiotemporal delta-gamma regression models to [DDX_sed_] to generate time-varying, spatially explicit predictions for sediment DDX concentrations across the SCB. We then applied Bayesian linear and linear mixed-effects models to understand the extent of coupling between sediment and fish DDX concentrations and how fish ecological traits, species, and year of collection impacted DDX bioaccumulation.

**Fig. 2.**
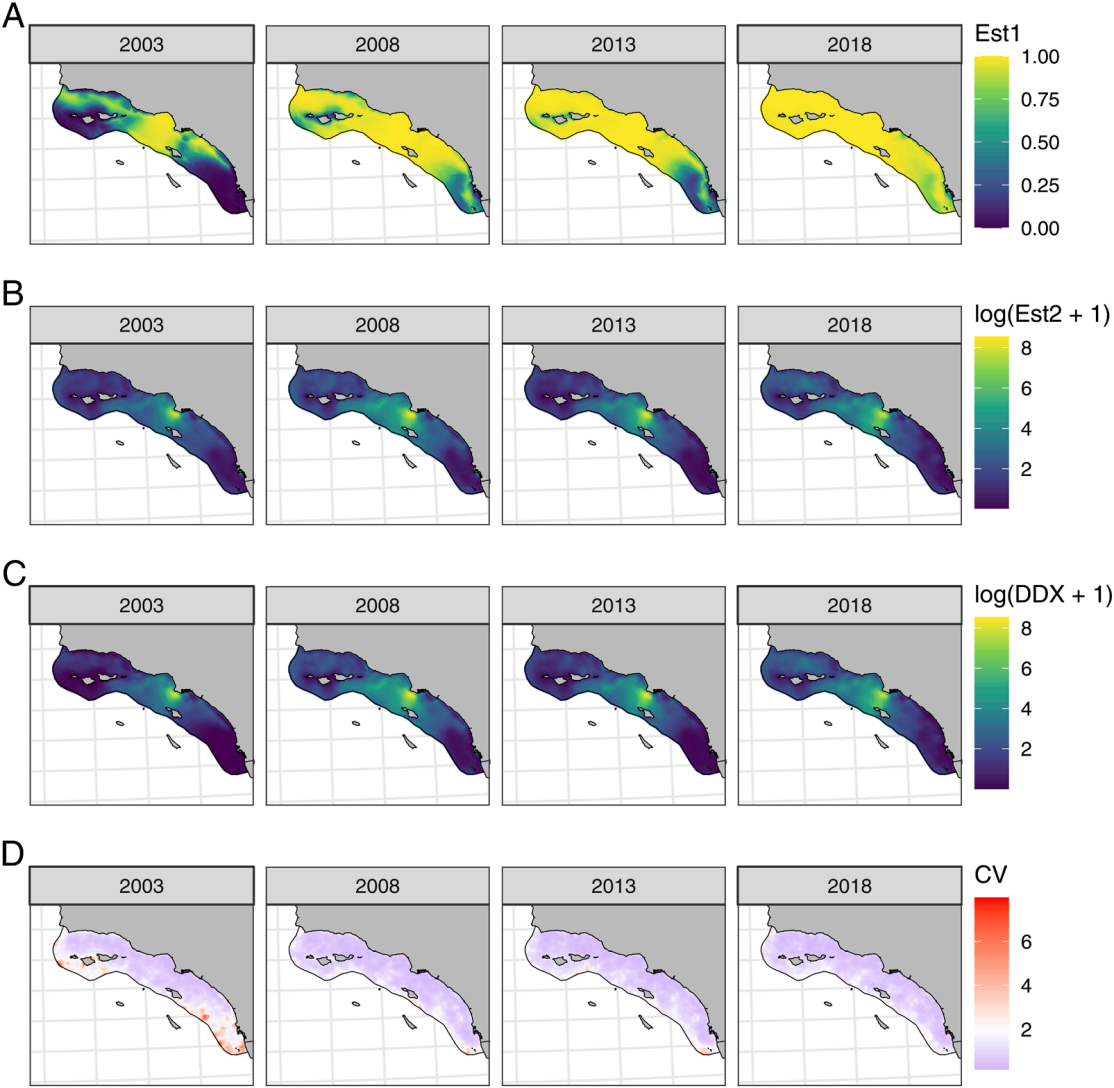
Sediment spatiotemporal model results by year showing the probability of detection via the binomial presence–absence model (Est1) (*A*), total DDX estimates from the gamma positive-value model (Est2) (*B*), total estimated DDX concentrations from both models (*C*), and the coefficient of variation on predictions (*D*).

Observed [DDX_sed_] ranged from 0.047 to 5,182.5 ng g^−1^ dw (56.72 ± 331.4 ng g^−1^ dw, mean ± SD), with 75% of samples showing detectable DDX concentrations. Ten percent of samples exceed the US National Oceanic and Atmospheric Administration's (NOAA) sediment quality guidelines (effects range median of 4,4′-(DDT + DDD + DDE) = 46.1 ng g^−1^ dw). The proportion of sediment samples with detectable DDX increased through time (Fig. 2 and *SI Appendix*, Fig. S3). DDX was detected in 56%, 72%, 76%, and 90% of samples from 2003, 2008, 2013, and 2018, respectively. The best-fit sediment regression model included spatiotemporal effects and both year and depth as covariates (*SI Appendix*, Table S4). Modeling results reflect changes in the number of sediment samples with detectable DDX, as the year coefficient for the binomial encounter model increased steadily through time (Fig. 2 and *SI Appendix*, Table S5) from −0.88 ± 0.87 (mean ± SE) in 2003 to 3.15 ± 0.90, 3.76 ± 0.90, and 5.57 ± 0.94 in 2008, 2013, and 2018, respectively. DDX-positive concentrations, on the other hand, were broadly similar across years. Year coefficients for the gamma concentration model were estimated to be 1.28 ± 0.50, 1.94 ± 0.50, 1.18 ± 0.50, and 1.62 ± 0.49 for 2003, 2008, 2013, and 2018, respectively.

[DDX_sed_] patterns generally showed expected gradients, wherein concentrations were highest near historic DDX disposal locations on the PVS (Fig. 2). Los Angeles Harbor and Santa Monica Bay tended to have higher DDX concentrations as well, with concentrations declining northward of the PVS. The lowest values were found offshore of San Diego. Spatial patterns for the encounter and concentration models were similar, with areas of higher concentrations having larger probabilities of encounter. Uncertainty was highest in deep, offshore locations (Fig. 2). These locations were less densely sampled, and therefore, prediction confidence was low as spatial interpolation occurred over a broad extent. The largest residuals occurred in ports, bays, and marinas, particularly San Diego Bay (*SI Appendix*, Figs. S4 and S5). These regions were intensively sampled and spatially heterogeneous, with very high [DDX_sed_] values occurring in close proximity to nondetect samples.

For nonlipid normalized fish composites, DDX concentrations ranged from 0.011 to 7,240.0 ng g^−1^ wet weight (16.2 ± 701.6 ng g^−1^ ww, mean ± SD) and lipid-normalized fish tissue DDX concentrations, [DDX_fish_], ranged from 0.015 to 5,583.3 ng 0.01 g^−1^ lipid (16.1 ± 466.1 ng 0.01 g^−1^ lipid, mean ± SD). Nearly all samples (93%) showed detectable DDX concentrations. Eighty-six percent of composites fell below the California EPA’s Office of Environmental Health Hazard Assessment least restrictive threshold of consumption for DDX (not to exceed seven servings per week, 220 ng g^−1^ ww), and 3% exceeded the most restrictive threshold of consumption (do not consume, 2,100 ng g^−1^ ww; *SI Appendix*, Table S6; ref. 36). There was a positive, linear association between transformed [DDX_sed_] and [DDX_fish_] in the null model with a mean slope of 0.53 and a highest posterior density 80% credible interval of [0.51, 0.55] and an intercept of 1.74 [1.67, 1.81] (*SI Appendix*, Fig. S6). In all cases, including unique effects by diet, habitat, or species improved model performance compared to applying a single slope and intercept between sediment and fish DDX (Table 1).

**Table 1.** Results of model selection for fish linear and mixed-effects models, showing model name, structure, LOOIC (leave-one-out information criterion), and elpd_loo_

Question	Model Name	Fixed effects	Random effects	LOOIC (SE)	elpd_loo_ (SE)	R^2^ (112)
1. What is the global relationship between [DDX_sed_] and [DDX_fish_]?	Null	[DDX_sed_]		3,518.3 (57.7)	−341.4 (25.3)	0.52
2. How do ecological factors mediate the relationship between [DDX_sed_] and [DDX_fish_]?	Diet (intercept only)	[DDX_sed_] + Diet		3,377.1 (55.5)	−270.8 (22.0)	0.59
	Diet (slope only)	[DDX_sed_] + [DDX_sed_]:Diet		3,356.5 (55.1)	−260.6 (21.5)	0.59
	Diet	[DDX_sed_] *Diet		3,342.9 (55.5)	−253.8 (21.5)	0.60
	Habitat (intercept only)	[DDX_sed_] + Habitat		3,364.6 (58.3)	−264.6 (23.7)	0.58
	Habitat (slope only)	[DDX_sed_] + [DDX_sed_]:Habitat		3,286.2 (59.3)	−225.4 (23.2)	0.61
	Habitat	[DDX_sed_]*Habitat		3,288.2 (59.3)	−226.4 (23.2)	0.61
3. Do additional factors improve prediction of [DDX_fish_]?	Diet- Habitat	[DDX_sed_] *Diet + [DDX_sed_] *Habitat		3,136.0 (55.3)	−150.3 (16.4)	0.67
	Diet-Habitat-Year	[DDX_sed_] *Diet + [DDX_sed_] *Habitat + Year		3,014.2 (54.6)	−89.4 (13.5)	0.70
	Diet- Habitat-Species	[DDX_sed_] *Diet + [DDX_sed_] *Habitat	1|Species	2,983.0 (61.3)	−73.8 (12.5)	0.72
	Diet-Habitat-Species-Year	[DDX_sed_] *Diet + [DDX_sed_] *Habitat + Year	1|Species	2,835.4 (58.1)		0.75

LOOIC describes the support of each candidate model where lower values indicate better models and the difference elpd_loo_ was used to compare model predictive capacity relative to the best model. For all models, [DDX_fish_] was treated as a left-censored variable, where values were constrained to fall between zero and the composite-specific MDL.

**Fig. 3.**
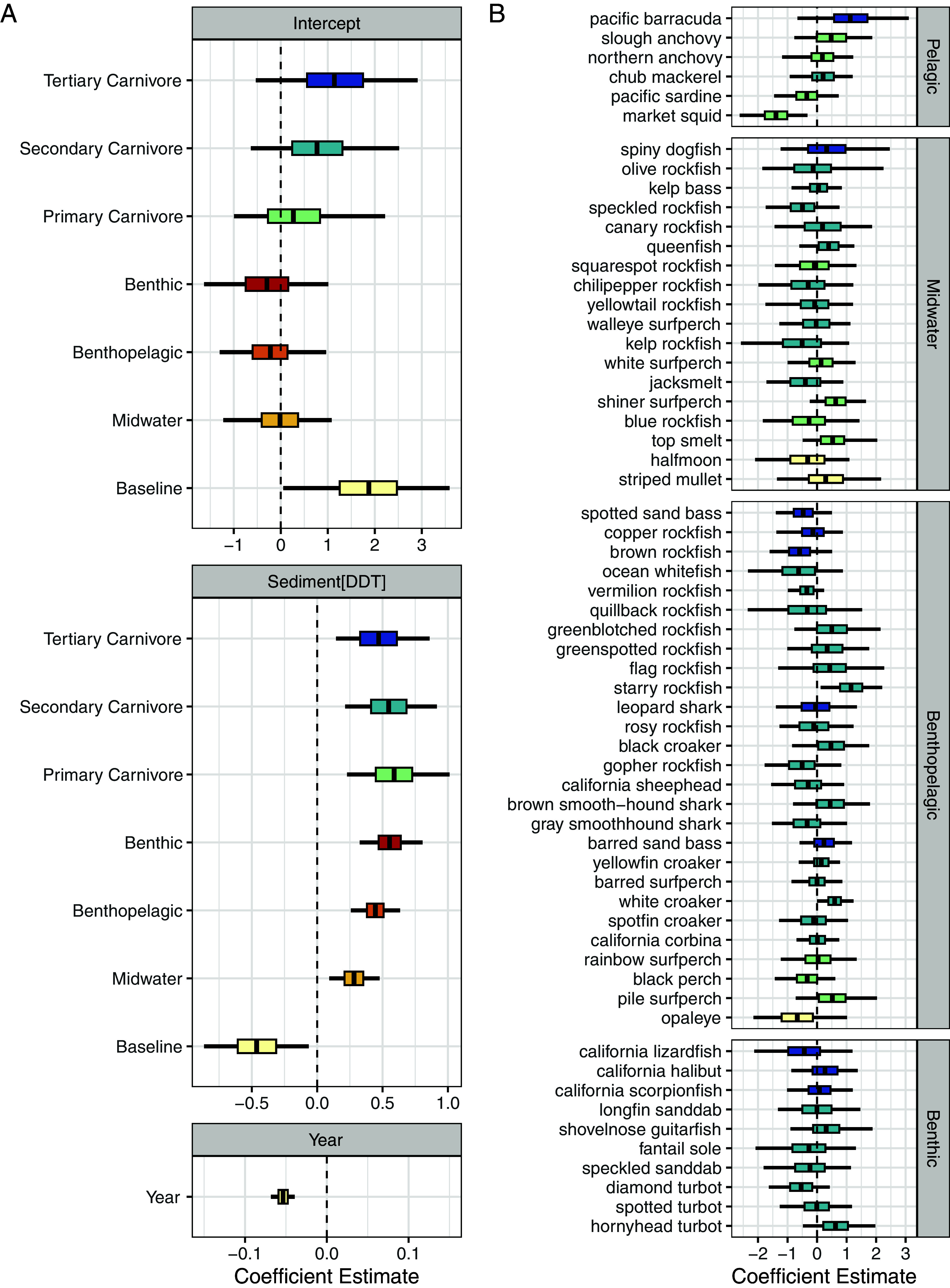
Model estimated posterior distributions for fixed (*A*) and random (*B*) effects from the diet-habitat-species-year model (Table 1). The reference category is herbivore and pelagic. Center lines are the mean estimated parameter, colored boxes represent the 80% credible interval, and the black lines are the maximum and minimum for each parameter distribution. Random effects (*B*) can be thought of as deviations from the group means. Color indicates diet categorization, with darker colors indicating higher trophic levels, and horizontal breaks denote habitat classification. Within each habitat category, species are arranged by the estimated FishBase trophic level.

The relationship between [DDX_sed_] and [DDX_fish_] varied by both diet and habitat, and the best-performing model of [DDX_fish_] included diet, habitat, year, and a random intercept for species (Fig. 3 and Table 1). Slopes varied substantially between midwater (0.28 [0.21, 0.35]), benthopelagic (0.45 [0.38, 0.51]), and benthic (0.55 [0.47, 0.64]) groupings but were generally less certain and more similar across primary consumer, secondary consumer, and tertiary consumer groups (0.59 [0.45, 0.73], 0.55 [0.41, 0.68], 0.47 [0.33, 0.61], respectively). Intercept estimates, on the other hand, varied across all groups. Estimates increased across primary, secondary, and tertiary carnivores (0.27 [−0.25, 0.83], 0.77 [0.24, 1.31], 1.14 [0.56, 1.75], respectively) and decreased across midwater, benthopelagic, and benthic groups (−0.02 [−0.41, 0.37], −0.22 [−0.60, 0.14], −0.29 [−0.75, 0.16], respectively). Based on the inclusion of species as a random effect, the data support that species-specific effects not represented by diet or habitat classification impact fish DDX bioaccumulation. Random intercept estimates ranged from −1.37 [−1.74, −1.01] for market squid to 1.15 [0.77, 1.53] for starry rockfish but were generally small and near zero (Fig. 3). The coefficient on year was negative (−0.053 [−0.06, −0.05]), indicating a decrease in [DDX_fish_] through time that was not explained by changing [DDX_sed_] values.

## 2. Discussion

**Fig. 4.**
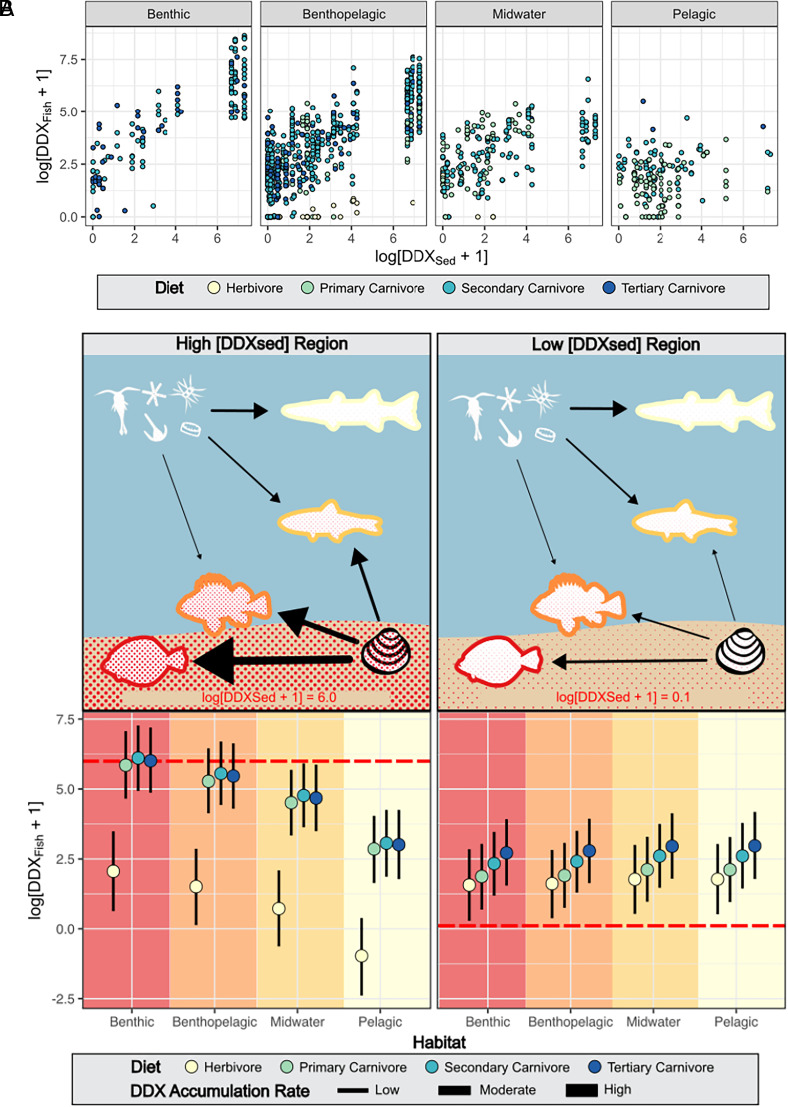
(*A*) [DDX_sed_] estimates for each fishing zone plotted against empirical [DDX_fish_] values, separated by diet (color) and habitat (position). (*B*) The top panel presents a conceptual figure of fish DDX bioaccumulation as a function of habitat, where red shading represents DDX burden and arrow size indicates DDX transfer from diet items. The bottom panel shows the 80% posterior predictive distribution of [DDXfish] for a highly contaminated (high [DDXsed]) and relatively pristine (low [DDXsed]) region. The horizontal red line in *B* indicates the [DDX_sed_] value used to generate predictions.

Despite more than half a century since the cessation of industrial dumping in the SCB, local ecosystem contamination continues to mirror the spatial legacy of DDX disposal. Thus, the impacts of ocean dumping on marine ecosystems and fisheries resources remain place-based, despite the long-held belief that the vastness of the ocean holds nearly limitless capacity for contaminant mitigation via dilution and advection (37). That said, our findings clearly demonstrate that ecological characteristics mediate the strength of linkages between sediment DDX concentrations and the DDX burdens of fishes. Based on our findings, we propose a conceptual model of DDX transport in the coastal ecosystem (Fig. 4) where the magnitudes of sediment signatures are highly conserved across space, and trophic ecology and habitat use mediate the vertical transport, lateral mixing, and biomagnification of DDX through the food web. Overarching all these factors, the magnitude of DDX in the SCB ecosystem as represented by fishes decreased over our study period, suggesting that the deleterious effects of these legacy pollutants in the SCB will continue to diminish over time as sediment DDX becomes less bioavailable due to degradation and burial.

### 2.1. Sediment DDX Is the Primary Predictor of DDX Burdens for Fishes.

A linear relationship between transformed [DDX_sed_] and [DDX_fish_] exists within the SCB across two decades and more than 60 species of fish. We provide evidence that local [DDX_sed_] is the primary control on [DDX_fish_] once lipid content of the fish composite has been accounted for and explains nearly half of the total variation in [DDX_fish_]. Results mirror previous, more targeted, studies from the region that found that contaminant concentrations of flatfish were highest near Palos Verdes where sediment concentrations maxima are located (32, 38) and SCB-wide relationships between sediment contaminant concentrations and flatfish tissue concentrations were highly correlated for both DDX and polychlorinated biphenyls (39–41). This research expands upon previous work and suggests that sediment can serve as a robust predictor of fish organic contamination across the full range of recreationally fished species.

The spatial distribution of DDX in sediments reflects historical disposal and ocean transport. The highest concentrations of DDX occurred on the PVS and immediately upcoast, presumably due to the discharge of roughly 870 to 1,450 tons of DDX onto the PVS between 1947 and 1971 (42) and the subsequent transport of these contaminants by ocean currents flowing northward at the discharge depth (43). The year coefficient of our encounter model systematically increased through time, likely due to a decrease in Method Detection Limits (MDLs) as analytical methods improved (*SI Appendix*, *Supporting Text* and Figs. S9 and S10) (44), yet we observed no systematic changes in nonzero [DDX_sed_] values throughout the sampling period. The lack of an observable trend in nonzero [DDX_sed_] values can potentially be attributed to sediment sampling procedures, where a single grab sample consisted of the top 2 to 5 cm of the sediment column. Sedimentation rates within the region have been recorded at 1 to 5 mm/y (27, 45) and natural degradation of DDX in marine sediments is slow, with reported half-lives generally >20 y (46, 47). Observed [DDX_sed_] values, therefore, represent a time-integrated average that would not be expected to change substantially over a 15-y sample period (48). Interestingly, although [DDX_sed_] showed limited change through time, [DDX_fish_] showed a marked decrease over the past 20 y, as evidenced by the negative year coefficient (Fig. 3; ref. 49). The most likely cause of this discrepancy is a decrease in the bioavailability of DDX to fish and benthic organisms not reflected in bulk [DDX_sed_] values, either due to downward migration of DDX in the sediment column via burial by clean sediments or reduced bioaccessibility due to contaminant aging (46). Although it is possible that changing fish population characteristics (e.g., fishing down populations to younger individuals) or diets (e.g., where or what fish are eating) resulted in the observed trends, it is unlikely given the consistent declines observed across sampled organisms.

### 2.2. Ecological Traits Mediate the Relationship between Sediment and Fish DDX Concentrations.

Although it’s commonly assumed that ecological traits play a consistent role in mediating organism contaminant exposure and bioaccumulation (50), our findings provide evidence that critical factors influencing [DDX_fish_] are context-dependent and contingent on [DDX_sed_] (Fig. 3). In our data, habitat emerged as a more important predictor of [DDX_fish_] and primarily impacted the slope term in linear model fits, whereas diet primarily modified the intercept term. Small differences in slope result in large differences in [DDX_fish_] at higher [DDX_sed_] values, whereas intercept term impacts are highest in low [DDX_sed_] environments. Therefore, in relatively pristine (low contaminant) environments, the primary predictor of fish bioaccumulation is trophic magnification. In highly contaminated areas, however, habitat becomes a more important predictor of DDX burdens (Fig. 4; ref. 40). We posit this occurs due to the relative balance of DDX transport from sediments to fishes (vertical transport), the spatial extent of contamination an organism integrates over (lateral mixing), and bioaccumulation due to trophic interactions (biomagnification) at a given sediment concentration.

DDX bioaccumulation increased faster with sediment concentrations for demersal species than for midwater and pelagic species residing higher in the water-column, as evidenced by the larger [DDX_sed_] slope term for benthic and benthopelagic fishes. Close correspondence between [DDX_sed_] and demersal species [DDX_fish_] can be explained by the hydrophobic nature of DDX. Due to its high octanol water partition coefficient, DDX does not readily dissolve in water and instead binds to suspended particles, organic matter, and sediments (8), resulting in sediment concentrations that can be orders of magnitude above water-column concentrations (23, 51). Demersal organisms exposed to these higher concentrations may bioaccumulate DDX to a greater extent through direct bioconcentration of DDX (52), incidental ingestion of contaminated sediment while feeding (53), or consumption of benthic food resources that are high in DDX due to the aforementioned processes (*SI Appendix*, Table S2). For fish, the most likely route of exposure is through diet (13, 14), and varying correlations of [DDX_sed_] with tissue concentrations across habitat groups is therefore likely reflective of differences in dietary pathways.

In addition to diet, the extent of lateral mixing further impacts fish DDX bioaccumulation. Demersal fish typically occupy a smaller range than pelagic or midwater species, and thus, their DDX concentrations are more reflective of local conditions (*SI Appendix*, Table S2). Benthic fish show both the largest slope term and smallest intercept term of any habitat group within our model, indicating they closely track local sediment conditions. For pelagic species that are characterized by seasonal migrations and extensive spatial movement (54–56), the region of capture may not be representative of where they forage and observed DDX concentrations may reflect a region-wide average of water-column DDX concentrations rather than local sediment concentrations.

Finally, biomagnification, a central process in ecotoxicology (57), was evidenced in our system by larger intercept estimates for higher trophic level organisms (Fig. 3). Consistent slope terms across primary, secondary, and tertiary consumers indicate similar rates of biomagnification across [DDX_sed_] gradients. Notably, patterns in biomagnification became evident only after accounting for sediment contamination. There is a long history of using trophic position as a predictor of organism contamination (58–60). Although terrestrial taxa show clear patterns of biomagnification (61), patterns among marine fishes are less clear, particularly when looking over a broad region or multiple species (20, 62). More localized studies tend to find biomagnification for DDX chemicals, however, exact rates can vary with latitude, chemical constituent, and subset of the food web sampled (57, 63). Recent modeling studies and reviews have examined the impact of variable contaminant exposure on bioaccumulation (64–66), however, empirical evidence for this process has been limited to date. Our study illustrates the importance of accounting for spatially variable contaminant exposures using empirical data, and results suggest that pooling samples across regions without accounting for background contamination is ill-advised.

Recognition of context-dependent controls on bioaccumulation is a substantial step forward in understanding of this issue, as previous results examining the relative importance of ecological factors on contaminant bioaccumulation have been mixed. Some studies found that foraging habitat was a stronger predictor of tissue contaminant concentrations and that the subsequent biomagnification was secondary (67–69) while others found that age and trophic position, as opposed to habitat and carbon source, dominated bioaccumulation (70, 71). Similar to our results, Dromard et al. (72) posited that biomagnification became a less important pathway of bioaccumulation in more contaminated areas and Fonseca et al. (73) found that rates of biomagnification were similar across sites with variations in biota concentrations reflecting baseline differences in site environmental levels.

### 2.3. Species-Specific Characteristics Play an Observable, If Limited, Role.

[DDX_sed_], diet, and habitat explained most variation in [DDX_fish_], however, species-specific factors, reflected in random effects, further impacted [DDX_fish_] (Table 1). Deviations from group means (i.e., species random effects) could reflect unmodeled aspects of life history such as growth, lifespan, or habitat utilization. For instance, species that grow quickly often exhibit low contaminant concentrations due to growth dilution, wherein fish add more tissue per unit contaminant consumed in prey, diluting contaminants in a larger biomass (74, 75). Growth dilution is perhaps evidenced by the large negative anomaly for Market squid (*Doryteuthis opalescens*), a pelagic species that lives only 9 mo (76), indicating lower-than-expected lipid-normalized DDX concentrations. Our modeling framework also implicitly assumes that fish composites reflect the distribution of sediment contaminants within their zone of capture; however, certain species preferentially use particular habitats within a given fishing zone, decoupling an individual’s exposure from the average benthic conditions (77, 78). For example, due to the documented positive relationship between depth, organic content of sediments, and [DDX_sed_] within the SCB (*SI Appendix*, Fig. S7), deeper dwelling fish that reside in organic-rich sediments, such as Starry rockfish (*Sebastes constellatus*), may accumulate more contaminants. Future research could use more specific location-of-capture information in conjunction with movement patterns to optimize sediment exposure calculations (79).

Our use of composites prohibited us from discerning how individual-level variation in sex (80, 81), diet (82, 83), or age (53) impacted fish DDX bioaccumulation. Although most monitoring programs used mature fish of legal size to ensure that analyzed specimens were representative of fish consumed by anglers (*SI Appendix*, Table S1), unaccounted for variation in fish composites still exist within our dataset. Nevertheless, our classification scheme provides a generalizable and accurate framework for predicting DDX burdens in fisheries species as a function of space and ecology, as demonstrated by the high predictive ability of our models. Understanding the interplay between life history, movement, and feeding patterns of an organism can help determine which exposure routes and stressors are likely to be most important to that particular species and should be the focus of future studies.

### 2.4. Implications of Predicting Contaminant Burdens in Coastal Fisheries.

Closer integration of ecotoxicology and ecology is essential for predicting contaminant impacts on biological communities and ecosystems (84, 85), and methods used here could be extended to other hydrophobic, bioaccumulative emerging and legacy pollutants to better inform animal and human exposure. Though bioaccumulation and sediment contamination are closely linked, they have typically been assessed separately (86). Our use of spatially and temporally distributed data from regional monitoring efforts offers a unique opportunity to assess the interplay among local sediment contamination, habitat use, and biomagnification in driving fish tissue contamination and highlights the tremendous value associated with multidecadal monitoring programs. Although our findings demonstrate that spatially explicit sediment measurements can serve as a robust predictor of fish contamination, they also clearly show that general ecological characteristics play an important role in determining the contaminant burden of any particular fish an angler may catch. Merging these pieces information in a predictive framework requires both monitoring data sufficient to characterize the spatial heterogeneity of contaminants and sufficient sample representation across ecological components of the community to parameterize a model of contaminant concentrations as a function of space and species life history. The advantage of such a prediction framework is that it can be generalized across species and life history, including those targeted by anglers, but with limited contaminant monitoring data.

Accurate and accessible consumption advisories are particularly pressing for vulnerable communities. Globally, fish consumption serves as a critical source of animal protein and micronutrients for low-income communities (87). Even in the SCB, many members of the angling community are socioeconomically disadvantaged (88) and rely on coastal fishing in a predominately urban context for food security (89, 90). The close association of sediment and fish DDX concentrations evident in our results implies that sediment could be used as a first-order proxy for fish contamination in regions of the globe without a current monitoring program, while knowledge of the general ecology and habitat of targeted species could help refine spatiotemporal consumption guidance. This contrasts with most current consumption advice that is generally based on species-specific sampling and contaminant assessments, and thus lacks generalization to unmeasured species and locations.

Our results indicate that using generalized ecological classifications produces results that are comparable to more detailed, species-specific models (Table 1). Most consumption advice to consumers generally occurs on a species-by-species basis (91), and consumption recommendations for unmeasured species can be absent or confusing. A more generalized framework leveraging ecological classifications paves the way for consumption advisories to be derived for unmeasured species. Furthermore, differing contaminant burdens across habitat groups have implications for how advisories are created. For example, a region-wide advisory for mobile pelagic species may be sufficient, whereas advisories for benthic fishes need to consider highly localized conditions. This integrated approach enhances our ability to address the complex dynamics of contaminant exposure and bioaccumulation, ultimately contributing to more informed environmental and public health policies.

Environmental contaminants are both increasing and diversifying through time, posing substantial threats to human and ecosystem health (2). Despite more than half a century since the cessation of industrial dumping in the SCB, the impacts remain prominent: DDX concentrations in marine mammals frequenting the region are among the highest worldwide (6) and critically endangered species, such as coastal populations of the California condor, are exposed to levels capable of impacting reproduction (92). Mitigating these impacts requires predictive tools that lay bare the routes of contaminant exposure through coastal ecosystems. Our findings provide a generalizable framework for predicting DDX burdens in fisheries species and suggest spatial and ecological nuance to the DDX ecosystem pathways of contaminant transport in the region. Our findings also support a cautionary approach to future ocean disposal of chemicals, where place-base impacts of dumping dominate the prediction of contaminant burdens in fisheries, decades, if not centuries, into the future. Leveraging this nuance should be an important part of efforts to safeguarding both people and the environment against the legacy of ocean dumping.

## 3. Materials and Methods

### 3.1. Datasets.

The area of focus of our study is the SCB (Fig. 1). Extending more than 600 km from the United States–Mexico border northward to Point Conception, California, the SCB is a dynamic and productive region where the cold, southward flowing California Current mixes with the warm, northward flowing Davidson Countercurrent (93, 94). The SCB is among the most biodiverse of all Northern Hemisphere temperate coastal ecosystems and one of the most densely populated coastal regions in the country, home to the nation’s largest commercial port, one of the largest US Naval complexes, and over 20 million people. Recreational marine fisheries in the region comprise a multibillion-dollar industry that is economically, socially, and culturally important (95, 96).

Laboratories that analyzed the sediment and fish data included in this study were subject to a common set of rigorous quality assurance and quality control guidelines to ensure comparability (44, 97). Sediment samples were collected via grab samples of the top 5 cm of sediment at embayment sites and the top 2 cm at offshore sites between July 1 and September 30 in 2003, 2008, 2013, and 2018 (Fig. 1 and *SI Appendix*, Fig. S3; refs. 24, and 34). Sites where concentrations were below detection limits were reported as zero values. For fish tissue, samples were collected off piers and boats and saved for analysis as either muscle tissue filet with the skin off (for large species) or whole fish without the head, tail, or internal organs (for small species). Generally, fish samples were composites of 5 to 10 specimens, depending on the species and monitoring program. We included only single-species composites in our analysis. Each fish composite was assigned to one of 27 spatially explicit fishing zones following McLaughlin et al. (32), however, composites from Jarvis et al. (98) were assigned to one of 25 spatially explicit California Department of Fish and Game 256 km^2^ fishing blocks (Fig. 1). Fishing zones and fishing blocks were combined into a common spatial array hereafter referred to as fishing zones. Assignment to fishing zones allowed us to approximate fish contaminant exposure when the exact capture location was unknown.

### 3.2. DDX in Sediment Modeling.

For each fishing zone, we generated a time-varying value for average sediment DDX concentrations, [DDX_sed_]. The goal with this analysis was not to determine environmental factors driving sediment DDX concentrations, but rather to generate zone-averages to be used as covariates in a model of fish bioaccumulation. To generate continuous spatial estimates for [DDX_sed_], we fit spatiotemporal regression models to sediment DDX measurements. The underlying statistical model was a spatiotemporal generalized linear mixed-effects model with Gaussian random fields to model spatiotemporal processes. Because concentrations were positive, skewed, and with frequent zero observations, we assume the data were observed with a delta-gamma distribution (99–101). The delta-gamma, or zero-modified gamma, distribution is the mixture of a gamma distribution with a positive probability mass at zero that separately models the probability of having nonzero values and positive values for each sampling event. It consists of a binomial presence–absence model (encounter model) and a model for positive values only (concentration model) with a gamma observation distribution and a log link.

We compared eight alternative models. Candidate models included a combination of spatial effects, spatiotemporal effects (first-order autoregressive spatiotemporal random fields to account for correlation from one time step to the next), year effects (as factors), and depth (modeled as a smooth function). We used a flexible spatial modeling approach as we expected [DDX_sed_] to be patchy due to an uneven legacy of dumping and the distribution of organic material within sediment, which covaries with depth within the SCB (*SI Appendix*, Fig. S7; ref. 24). We fit our models using the “sdmTMB” R package (102), which makes use of the integrated nested Laplace approximation (103) to generate stochastic partial differential equation matrices and Template Model Builder (104) to calculate the log-likelihood, gradient of the log-likelihood and implementation of the Laplace approximation. Candidate models were compared via ΔAIC (Akaike information criteria) and fivefold-cross-validation (*SI Appendix*, Table S4). The best-fit model was subsequently used to predict average [DDX_sed_] for each fishing zone corresponding to the four time periods captured by both sediment and fish data: 1998 to 2005, 2005 to 2010, 2010 to 2015, and 2015 to 2020.

### 3.3. DDX in Fish Modeling.

To understand the extent of coupling between sediment and fish DDX concentrations, we fit 11 candidate Bayesian linear and linear mixed-effects models to [DDX_sed_] and lipid-normalized fish DDX concentrations, [DDX_fish_] (Table 1). Prior to analysis, fish and sediment values were transformed as log(x + 1), where x is the DDX concentration, to account for right-skewness and zero values. In all models, [DDX_sed_] was treated as a continuous variable assuming normal errors. [DDX_fish_] was treated as a continuous, left-censored variable where nondetect values were constrained to fall between zero and the MDL. As MDLs were variable across analytes and monitoring programs, the minimum MDL across all six DDX analytes was selected for each fish composite.

First, to understand the mean relationship between [DDX_sed_] and [DDX_fish_], we included [DDX_sed_] as a fixed effect with no hierarchical groupings. Next, to understand whether and how fish ecological traits mediate this relationship, six additional models were fit to the same dataset. The first set examined the impact of fish diet on [DDX_fish_] values and consisted of three individual models. The first included the interaction between [DDX_sed_] and fish diet as a categorical variable (slope and intercept estimated for each group), the second included fish diet as a fixed effect (intercept only), and the third included only the interaction between [DDX_sed_] and fish diet (slope only). The second set of models took the same form but examined the relationship between [DDX_sed_] and fish habitat (as a categorical variable). This formulation allowed the estimation of unique slopes and intercepts between [DDX_sed_] and [DDX_fish_] across different diet or habitat classifications. Finally, we fit four additional models to understand whether species-specific variation and time further influenced [DDX_fish_] values. Models included both diet and habitat as interaction terms for [DDX_sed_], species as a random effect, and year as a continuous, centered variable.

Parameter estimates were obtained using the “brms” R package (105), which makes use of Stan (106) to implement a Hamiltonian Monte Carlo Sampler and its extension the No-U-Turn Sampler (107). Weakly informative priors were used for random effects (Cauchy distribution with a location of 0 and scale of 2) and fixed effects (Cauchy distribution with a location of 0 and scale of 1). Candidate models were compared via approximate LOOIC using the “loo” package (108), where the lowest value indicates the best model, and via the difference between theoretical expected pointwise predictive density (elpd_loo_) for each model compared to elpd_loo_ of the best model (Table 1). Diagnostics for candidate models can be found in *SI Appendix, Supporting Text*.

## Supplementary Material

Appendix 01 (PDF)

## Data Availability

Sediment and Fish DDX data have been deposited in FigShare (https://doi.org/10.6084/m9.figshare.25043819.v1) (35).
