# Supplementary material for: The persistent DDT footprint of ocean disposal, and ecological controls on bioaccumulation in fishes
Source: Proc Natl Acad Sci U S A. 2024 Oct 28;121(45):e2401500121. doi: 10.1073/pnas.2401500121 (PMC11551384; doi:10.1073/pnas.2401500121)
Supplement: Supplementary file 1 — Appendix 01 (PDF) [file pnas.2401500121.sapp.pdf]

PNAS

Supporting Information for

The persistent DDT footprint of ocean disposal, and ecological controls on bioaccumulation in fishes

Lillian McGill[a]*, Toni Sleugh[a], Colleen Petrik[b], Kenneth Schiff[c], Karen McLaughlin[c], Lihini Aluwihare[d], Brice Semmens[a]

[a]Marine Biology Research Division, Scripps Institution of Oceanography, University of California, San Diego, La Jolla, CA 92093
[b]Integrative Oceanography Division, Scripps Institution of Oceanography, University of California, San Diego, La Jolla, CA 92093
[c]Southern California Coastal Water Research Project, Costa Mesa, CA 92626
[d]Geosciences Research Division, Scripps Institution of Oceanography, University of California, San Diego, La Jolla, CA 92093

*Corresponding author
Email: lmmcgill@ucsd.edu

This PDF file includes:

Supporting text
Figures S1 to S11
Tables S1 to S6
SI References

Supporting text

Fish Model Fit. The relationship between [DDX_{sed}] and [DDX_{fish}] varied by both diet and habitat (Table 1 and SI Appendix, Fig. S6). For habitat classifications, the slope-only model and the model that included both slope and intercept effects were comparable and more supported than the intercept-only model. In the slope and intercept model, slopes varied substantially between baseline (pelagic) (0.10 [0.03,0.17], mean estimate [highest posterior density 80% credible interval]), midwater (0.27 [0.19, 0.35]), benthopelagic (0.42 [0.35, 0.50]), and benthic (0.56 [0.47, 0.64]) groupings. Intercept estimates showed less variability. Baseline (pelagic), benthopelagic, and benthic groupings had similar estimates (1.72 [1.54, 1.90], 0.10 [0.03, 0.17], 0.16 [0.03, 0.36], respectively), whereas the intercept estimate for the midwater group was slightly larger (0.31 [0.07, 0.53]).

For diet classifications, the most supported model included both slope and intercept effects. Diet-model coefficients exhibit a somewhat inverse pattern to the habitat-model. Intercept estimates varied across baseline (herbivore), primary consumer, secondary consumer, and tertiary consumer groups (1.21 [0.81, 1.58], 0.25 [-0.15, 0.66], 0.66 [0.28, 1.06], 0.78 [0.39, 1.20], respectively). Slope estimates were close to estimates from the null model for primary consumer (0.54 [0.38,0.70]), secondary consumer (0.68 [0.53,0.83]), and tertiary consumer (0.67 [0.52,0.83]) groups, however, the baseline (herbivore) group had a much lower slope estimate (-0.13 [-0.29, 0.02]).

We performed typical diagnostic analyses to confirm goodness of fit, identify residual patterns, and to evaluate overdispersion for our final best-fit model. We used a simulation approach and simulated data from the fitted model conditional on the fitted random effects. We modified functions in the DHARMa R package (1) and the DHARMa.helpers R package (2) to perform calculations on our censored “brms” model output wherein the simulated values were censored to zero if predicted concentrations fell below the minimum MDL for a given fish sample. The best-fitting model indicated no diagnostic issues (SI Appendix, Figure S8). The quantile plot indicated no systematic deviation away from the assumed distribution (Kolmogrov-Smirnoff p-value = 0.26). To test for overdispersion, we compared the observed variance of the residuals to simulated variance of the residuals, as implemented in the DHARMa and DHARMa.helpers packages. We found no evidence of overdispersion of the data from the fitted model ($p = 0.358$).

Sensitivity of [DDX_{sed}] fishing zone-averages to increasing MDLs. The Method Detection Limits (MDLs) for 2,4′-DDE, 4,4′-DDE, 2,4′-DDD, 4,4′-DDD, 2,4′-DDT, and 4,4′-DDT in sediment samples within the Southern California Bight (SCB) generally increased through time (SI Appendix, Fig. S9). The increased detection limits are reflected in the binomial encounter model year coefficients and spatiotemporal random fields, wherein encounter is more likely given low MDLs, as everything below the MDL is treated as a zero. We conducted a sensitivity analysis to determine whether changing MDLs affected zone-wide averages for [DDX_{sed}] that were used in subsequent fish models.

As MDLs were variable across analytes, for the sensitivity analysis we assumed a universal MDL of 1 ng g^{-1} (or zero on the log-scale) and censored all [DDX_{sed}] values below this threshold. This resulted in the conversion of 155 values from positive estimates to zeroes (N_{2003} = 5, N_{2008} = 33, N_{2013} = 58, N_{2018} = 59). We then ran the same sediment best-fit model (Model 8 in SI Appendix, Table S4 and SI Appendix, Fig. S10) and used output to predict the average [DDX_{sed}] for each fishing zone corresponding to the four sampling time periods. We compared zone-wide averages for our original data to the censored data (SI Appendix, Fig. S11).

Results indicated that censoring data using a higher MDL did not substantially alter zone-wide average sediment DDX concentrations, as most zones fell on or near the 1:1 line. Fishing zones in the southern region of the SCB (e.g., San Diego Shelf) showed the largest differences between censored and uncensored data, likely due to greater censoring of data from within this region due to lower overall [DDX_{sed}] values. As differences were small and general patterns preserved, we elected to use uncensored data within this paper.

Proportional analysis of DDX. We examined how the major DDX compounds, DDD, DDE, and DDT, were distributed across sediment and fish samples. For data with detectable DDX concentrations, DDE was the primary DDX compound present in both sediment and fish samples. For fish samples, an average of 97.1% of DDX was DDE, followed by DDD at 2.4% and DDT at 0.5% (SI Appendix, Figure S2). The trend is the same with sediment data, however, DDD and DDT were slightly more common. For sediments, the average DDE contribution to total DDX was 88.6%, followed by 7.9% for DDD and 3.5% for DDT (SI Appendix, Figure S2). Sediment data used in this analysis agrees with recent studies of the region, which have found that DDE is the primary DDX compound present in sediment on the PVS (3), whereas offshore stations between the PVS and Santa Catalina Island exhibit higher but variable proportions of DDT and DDD, especially within the most highly contaminated strata associated with offshore disposal (4).

We focused our analysis on total DDX, as statistically predicting contaminant compositions in fish based on sediment is challenging, and generally process-based models have been used to this end (5). Bioconcentration and biomagnification are influenced by several factors, with the octanol-water partition coefficient (log-Kow) being a key determinant. Higher log-Kow values generally lead to greater bioconcentration and biomagnification within the observed DDX ranges (6, 7). Taylor et al. (8), however, found an inverse relationship between bioaccessibility and log-Kow for DDX compounds and that bioavailability was impacted by organic matter content within sediments, as organic matter was considered to be the primary domain for binding hydrophobic contaminants (9). The relative importance of these two processes —enhanced bioaccumulation potential versus reduced bioavailability— cannot be easily resolved in a statistical framework. Our methods demonstrate that for bulk DDX, the value most often used to develop consumption advisories, a statistical relationship is sufficient to predict DDX concentrations in fish. Future work should leverage the compositional nature of the data, as summary metrics like “total DDX” encompass many tracers that, while correlated, contain unique information. Additionally, future studies could integrate fish and sediment data within a process-based modeling framework to address the complexities of contaminant uptake, although such work is beyond the scope of this study.

Supporting Information Figures

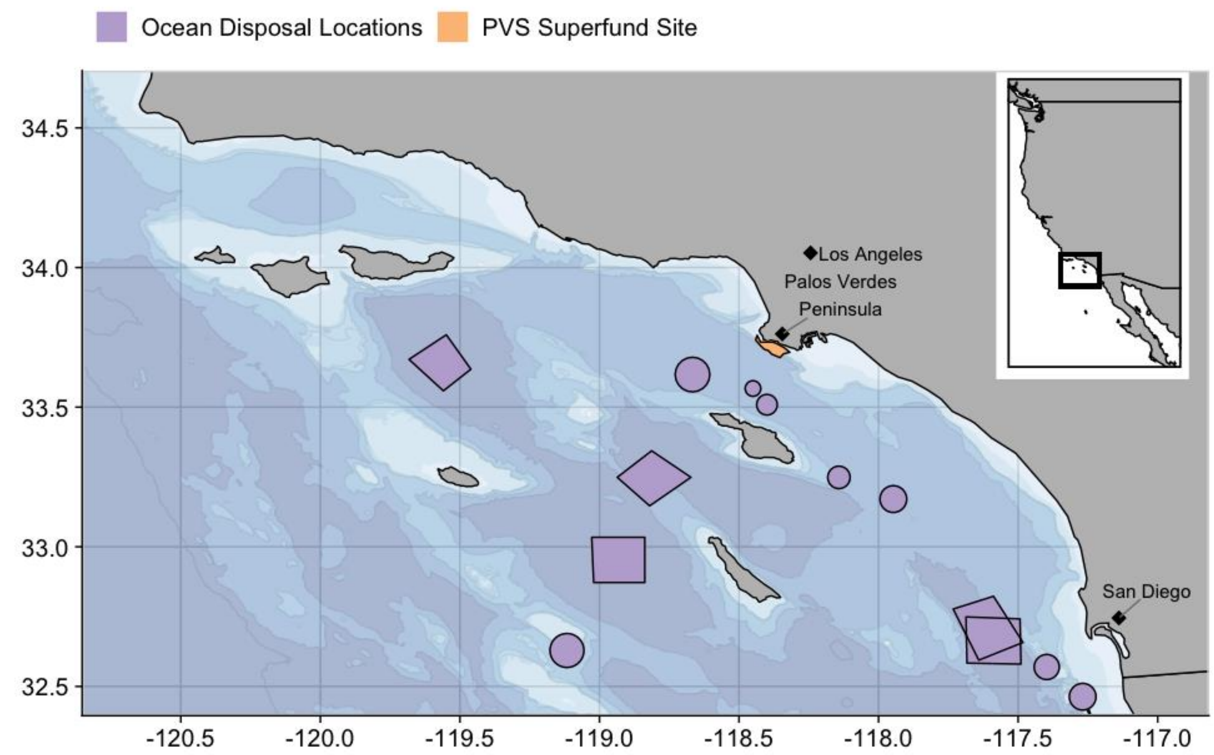

Fig. S1. Map of the Palos Verdes Shelf superfund site (orange polygon) and 13 known deep ocean disposal locations within our study area (purple polygons).

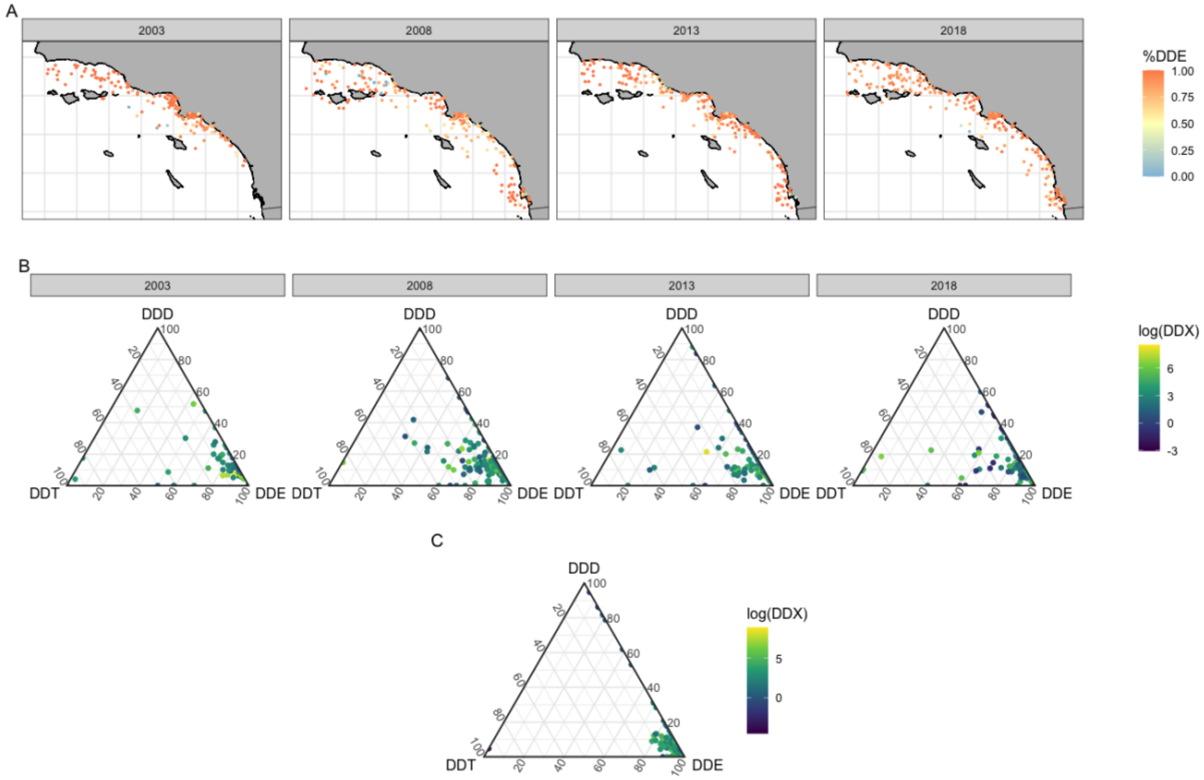

Fig. S2. Maps of the relative fraction of DDE in sediment samples (A) and ternary plots of the relative fraction of DDT, DDD, and DDE in sediment samples (B), separated out by year. Ternary plot of the relative fraction of DDT, DDD, and DDE in fish composites (C). For (B) and (C), symbol color represents the log concentration of total DDX.

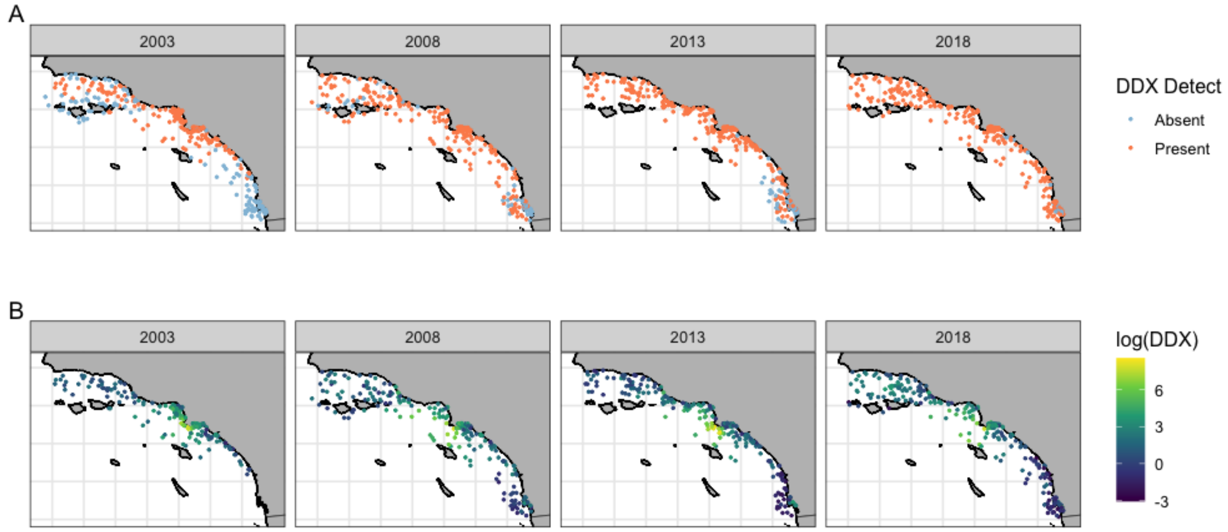

Fig. S3. Empirical data for sediment DDX presence (A) and log total DDX concentration (B), separated out by year.

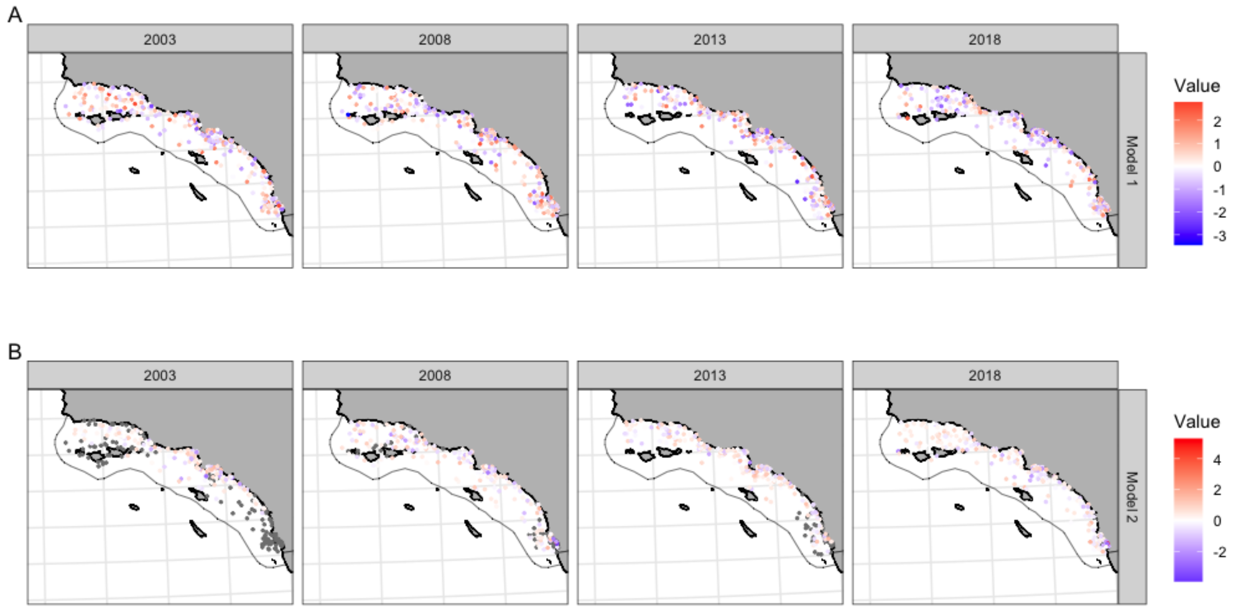

Fig. S4. Standardized quantile residuals of DDX concentrations from the most supported model for sediment DDX presence (A) and log total DDX concentration (B), separated out by year.

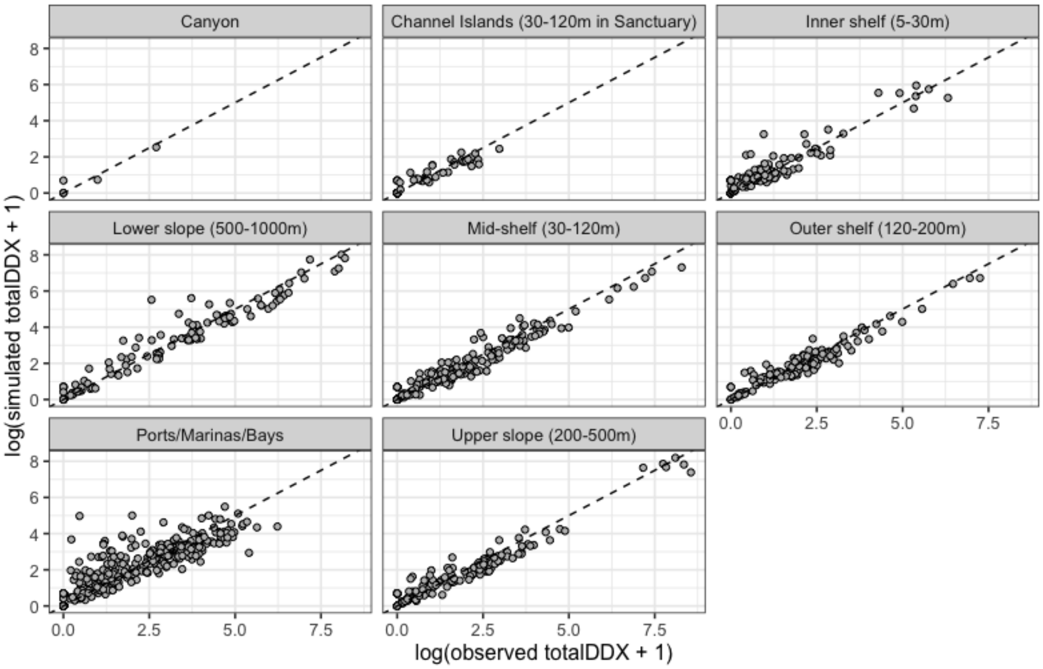

Fig. S5. Observed vs. simulated log total DDX concentrations for sampling sites, broken apart by stratum. Residuals are generally largest in the Ports, Marinas, and Bays stratum. This may be due to high physical heterogeneity across short distances (i.e., sharp increases or decreases in depth and organic material) and numerous localized inputs of organic contaminants (i.e., runoff, rivers, proximity to industry), which are not well represented by the model. Cross validation statistics are presented in Table S4.

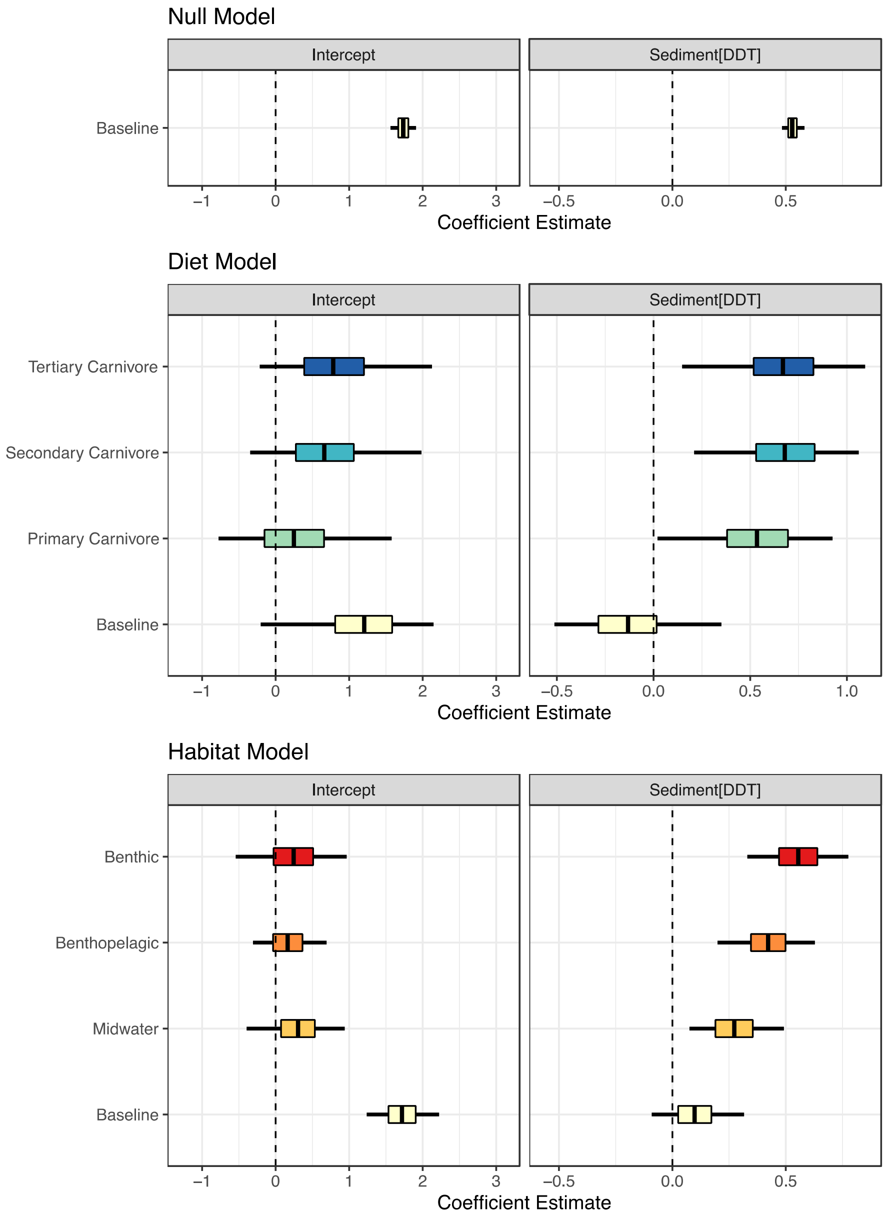

Fig. S6. Model estimated posterior distributions from the null-model (A), diet-model (B) and habitat-model (C; Table 1). The baseline category for the diet model is herbivore and the baseline category for the habitat model is pelagic. Points are the mean estimated parameter, colored boxes represent the 80% credible interval, and the black lines are the maximum and minimum for each parameter distribution. Model fits are shown in Table 1.

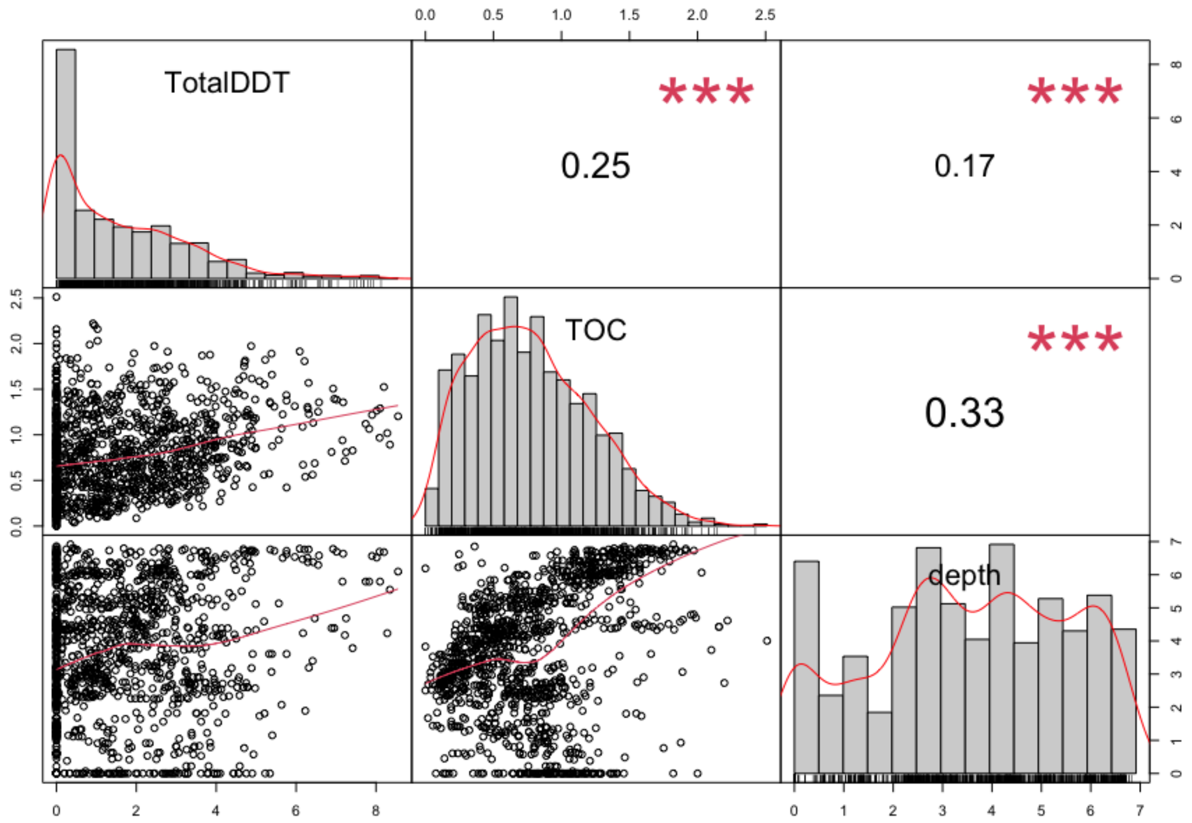

Fig. S7. Correlations between total DDX, total organic carbon (TOC), and depth across all years and stations. The top panels display the absolute value of the correlation between variables, the diagonal panels show the distribution of each variable, and the bottom panels display bivariate scatter plots and a fitted line. All values were transformed as log(x+ 1) prior to analysis.

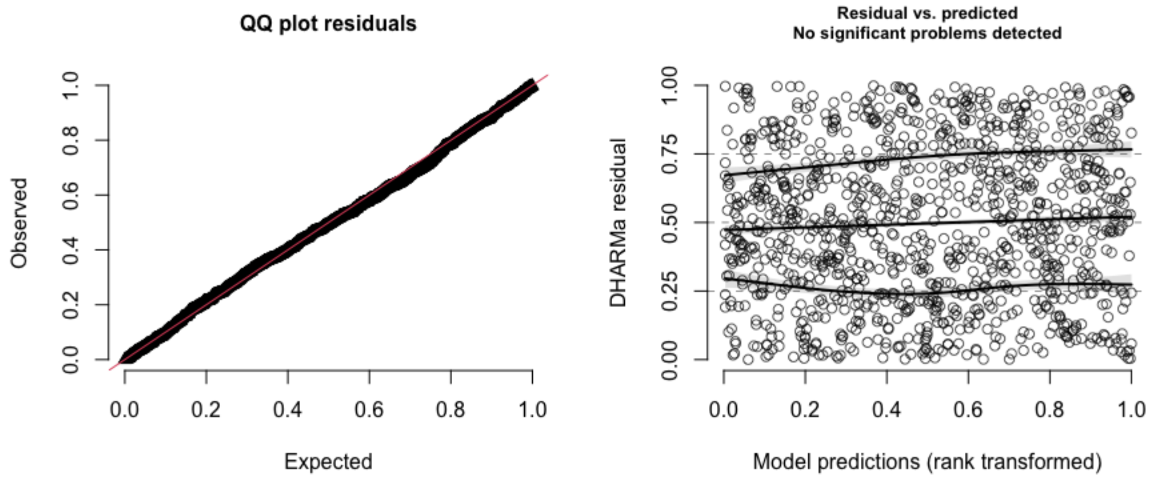

Fig. S8. Diagnostic plots of best-fitting model predicting [DDX_{fish}] as a function of [DDX_{sed}], diet, habitat, species, and year. Left panel depicts expected (theoretical) versus observed scaled residuals, where each point is an observation, and the red line indicates observed = expected. Right panel depicts the scaled residuals as a function of rank-transformed model prediction. The horizontal line at 0.5 is the expectation, the curved lines are quantile fits for observed data.

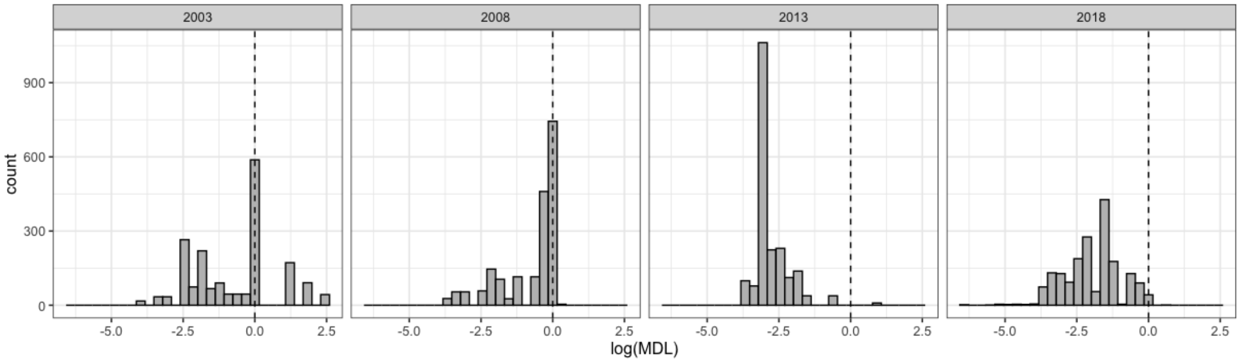

Fig. S9. Log-method detection limits through time for 2,4′-DDE, 4,4′-DDE, 2,4′-DDD, 4,4′-DDD, 2,4′-DDT, and 4,4′-DDT in sediment samples within the SCB. For the sensitivity analysis, we assumed a MDL of 1 ng g^{-1} (or zero on the log-scale, dashed line) and censored all [DDX_{sed}] values below this threshold.

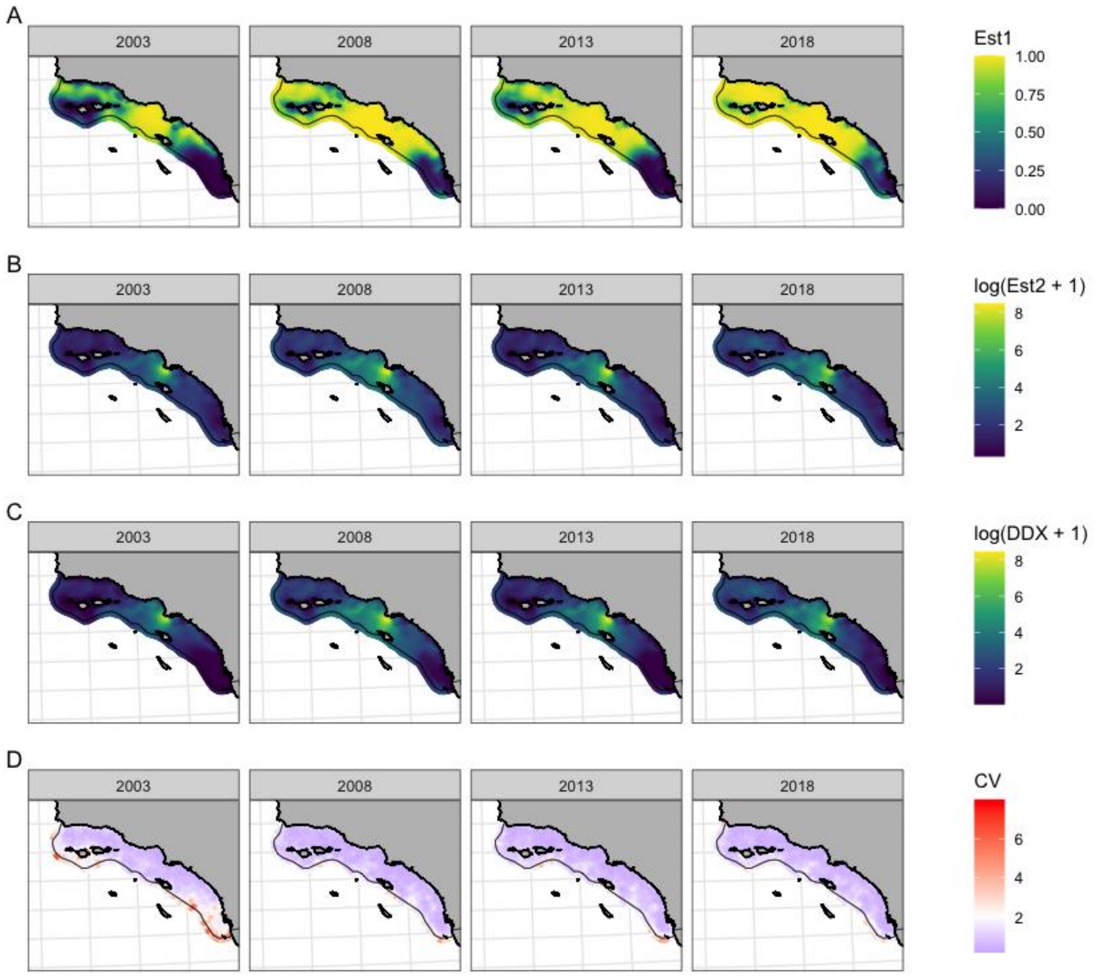

Fig. S10. Sediment spatiotemporal model results for censored data by year showing the probability of detection via the binomial presence-absence model (A), log total DDX estimates from the log-link gamma model (B), total estimated DDX concentrations from both models in ng g^{-1} dw (C), and the coefficient of variation on predictions (D).

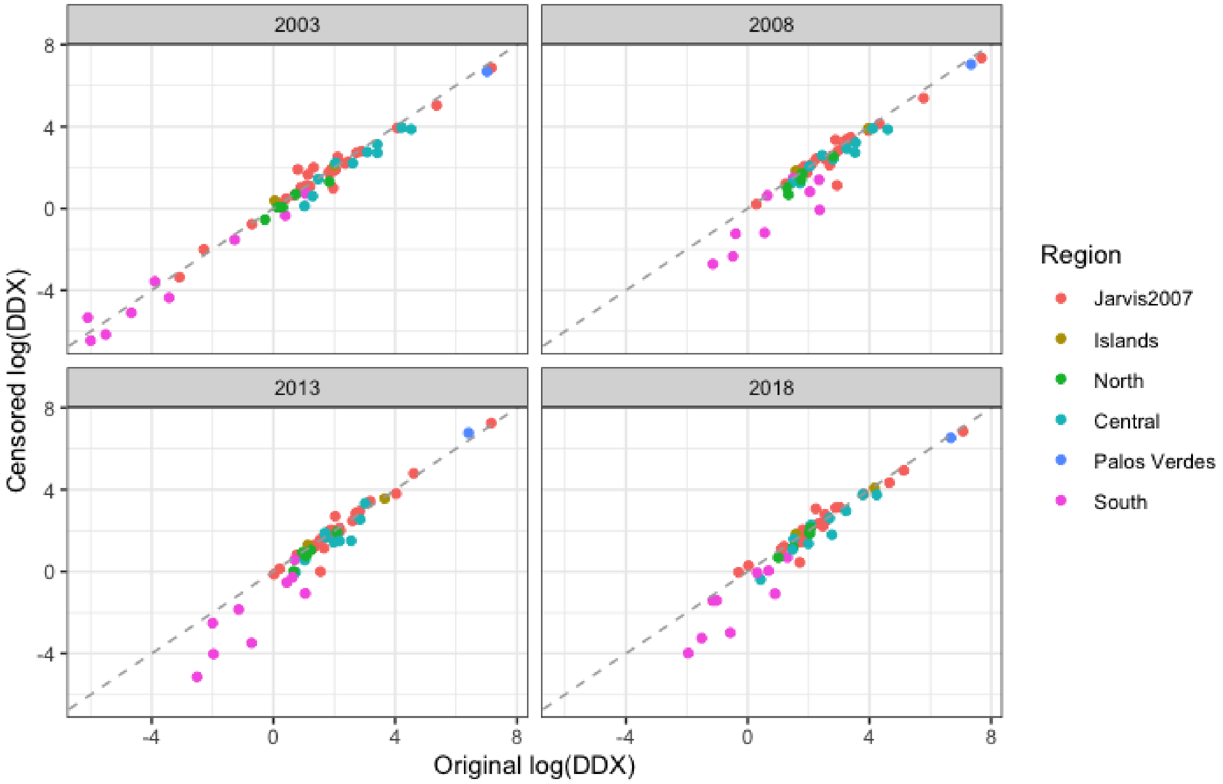

Fig. S11. Zone-wide averages for [DDX_{sed}] using original data (x-axis) and censored data (y-axis). Points are colored by fishing-zone region to better visualize spatial differences, and the dashed line indicates 1:1 correspondence.

Supporting Information Tables

Table S1. Inventory of data used in this study. The Southern California Bight (SCB) is defined as Point Conception, CA to US-Mexico border.

Data source	Data type	Sample #	Spatial extent	Temporal extent	Sample collection protocol
Southern California Bight Regional Monitoring Program (10)	Sediment chemistry and organic matter	1275	SCB	2003, 2008, 2013, 2018	The Southern California Bight Regional Monitoring Program uses a stratified random sampling design for sediment samples to ensure an unbiased sampling approach to areal assessments of environmental condition.
Statewide Coastal Screening Survey (11, 12)	Fish composite chemistry, total length (mean, maximum and minimum), sex, and species	142	Coastal CA, filtered to only SCB	2009-2010, 2018-2019	Fish of legal size or larger were preferred but not required. If more than five specimens were collected, then the middle 75% of the length distribution was used for the composite. Fish from this interquartile range were selected at random for inclusion in each composite.
Coastal Fish Contamination Program (13)	Fish composite chemistry, total length (mean, maximum and minimum), sex, and species	274	Coastal CA, filtered to only SCB	Annually from 1998-2003	None specified.
Jarvis et al. 2007 (14)	Fish composite chemistry, total length (mean), and species	88	Pelagic SCB	2003-2004	Specimens were sampled at random from commercial landing markets and bait receivers, who obtained specimens from commercial purse-seine fishing vessels.
McLaughlin et al. 2021 (12)	Fish composite chemistry and species	145	SCB	2018-2019	Fish of legal size or larger were preferred but not required. If more than five specimens were collected, then the middle 75% of the length distribution was used for the composite. Fish from this interquartile range were selected at random for inclusion in each composite.
Southern California Coastal Marine Fish Contaminants Survey (15)	Fish composite chemistry, total length (mean, maximum and minimum), and species	90	Palos Verdes, Santa Monica Bay, and San Pedro Bay regions	2002-2004	Fish size ranges were specified based on the middle 80 percent of reported fish lengths in the Pacific States Marine Fisheries Commission's recreational fishing database (RecFIN). All fish kept were within the State of California Department of Fish and Game legal collection limits.

Los Angeles County Sanitation Districts Local Trends Assessment (16)	Fish composite chemistry, standard length (maximum and minimum), and species	111	Adjacent to White Point sewer outfall	Annually from 2006-2021	Ten consistent sized specimens of target species fish were collected from each zone via otter trawl.
Los Angeles County Sanitation Districts Seafood Safety Assessment (16)	Fish composite chemistry, standard length (maximum and minimum), and species	93	Adjacent to White Point sewer outfall	2006, 2008, 2010, 2012, 2014, 2016, 2018, 2020	The target size range for each species is based upon applicable legal-size limits for the species, examination of available size frequency distribution data for the region, and USEPA guidance (USEPA 2000).
City of San Diego Ocean Monitoring Program (17)	Fish composite chemistry and species	131	Adjacent to Point Loma and South Bay sewer outfalls	Annually from 2001-2022	Only fishes with standard lengths ≥ 11 cm were retained to ensure the collection of sufficient tissue for analysis, while minimizing total catch necessary.

Table S2. Inventory of fish species used in this study. Fish photos are from the California Department of Fish and Wildlife (https://marinespecies.wildlife.ca.gov/). Much of the literature on rockfish and bottom species summarized below was collated by the Pacific Coast Groundfish Fishery Management Plan (18). Fish species were classified into four categories for both diet (herbivore, primary consumer, secondary consumer, tertiary consumer) and habitat (benthic, benthopelagic, midwater, pelagic). Diet categories represent coarse approximations of trophic level for each species. This classification scheme builds off of diet classifications developed by subject matter experts for the Southern California Bight (19) that were available for 30 of the 61 species included in our analysis. Whenever possible, we deferred to this scheme for trophic classifications, and when unavailable, we used a combination of empirical diet studies from the SCB and California Current and FishBase estimated trophic level to assign diet categories.

	Species Name (sample number)	Scientific name	Diet Classification	Habitat Classification
1	Barred sand bass (60) 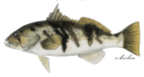	*Paralabrax nebulifer*	**Tertiary carnivore;** Adult barred sand bass consume fish, octopus, crabs, polychaete worms and ascidians. Roberts et al. (20) examined the feeding habits of 165 individuals from Southern California and found brachyuran crabs, mysids, pelecypods, and epibenthic fishes were the most important prey, with larger fish relying more heavily on fish and cephalopods. An additional diet study found that adult barred sand bass feed primarily (90% by weight) on benthic and epibenthic sand and reef associated fishes, with 45% of their total diet consisting of reef species and 55% from sand associated species (21). Horn and Ferry-Graham (19) classify barred sand bass as a tertiary carnivore among inner shelf fishes and Allen (22, 23) defined the feeding guild as bottom-living ambushing benthopelagivore. FishBase estimates a trophic level of 3.5.	**Benthopelagic**; Barred sand bass are a benthic species (24) that inhabit the ecotone, which is the transitional habitat where sand or mud bottom meets coastal rocky reefs. They show a preference for sand habitat within close proximity to reefs and rock habitat (21, 25, 26). As a result, barred sand bass belongs to the "shallow rock sand" assemblage based on relative abundance and co-occurrences of species within habitats (27). They tend to exhibit site fidelity, with an average home range size of 10 km, but may migrate in the late spring to mid-summer to form spawning aggregations (26). Primary depth range is 0-40 m and potential for adult dispersal is moderate.
2	Barred surfperch (33) 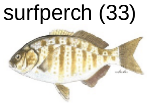	*Amphistichus argenteus*	**Secondary carnivore;** The diets of barred surfperch consist primarily of benthic invertebrates such as the Pacific mole crab (*Emerita analoga*), amphipods, skeleton shrimps, and polychaete worms. They also will consume smelt embryos and small fish such as Pacific sardine and northern anchovy (28). One study found that Pacific mole crab dominate the diet of adult barred surfperch, accounting for 92% of their diet, with limited contributions from intertidal clams and amphipods (29). FishBase estimates a trophic level of 3.5.	**Benthopelagic**; Barred surfperch are found in the surf on open coast sandy beaches and in bays and estuaries (30, 31) and are classified as belonging to the "surf zone and nearshore soft bottom" assemblage based on relative abundance and co-occurrences of species within habitats (27). Potential for adult dispersal is moderate.
3	Black croaker (5) 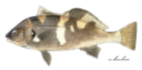	*Cheilotrema saturnum*	**Secondary carnivore;** Black croaker likely share a similar diet to the white croaker, including small crabs, shrimp, and other crustaceans. Fish base estimates a trophic level of 3.6.	**Benthopelagic;** Black croaker is a bottom-associated species (24) classified as belonging to the "nearshore nocturnals" assemblage based on relative abundance and co-occurrences of species within habitats (27). Primary depth range is 0-45 m and potential for adult dispersal is low.
4	Black perch (55) 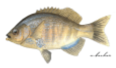	*Embiotica jacksoni*	**Primary carnivore;** Horn and Ferry-Graham (19) classify black perch as a primary carnivore among rocky reef and kelp bed fishes. Black perch primarily rely on benthic invertebrates. Allen (22) found the most important prey items to be gammaridean amphipods and reptantian decapods. Polychaete worms, shrimp, and gastropods also occurred commonly in stomachs. Across other studies, amphipods, caprellids, polychaete worms, and crabs have generally been found to be the most important prey (32–34). An analysis of 111 fish from Southern California (35) supports these findings. The diet database shows that on average, 62% of the diet by	**Benthopelagic**; Black perch is a bottom-associated species (24) classified as belonging to the "shallow rock sand" assemblage based on relative abundance and co-occurrences of species within habitats (27). The species is normally associated with rocky habitats covered with turf algae (36) and feeds along rocks and the sand-rock interface inshore to 1.5 m (34). During the day most fish are suprabenthic or midwater (22, 36). Potential for adult dispersal is moderate.

	Species Name (sample number)	Scientific name	Diet Classification	Habitat Classification
			volume was composed of amphipoda, followed by under 5% of echinodermata, decapoda, benthic arthropods, benthic worms, and tunicates. Allen (22, 23) defined the feeding guild as water-column cruising diurnal benthopelagivore. Fish base estimates a trophic level of 3.2.	
5	Blue rockfish (2) 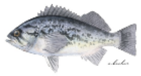	*Sebastes mystinus*	**Primary carnivore;** McCain et al. (18) states that tunicates, hydroids, jellyfishes, salps, crustaceans such as krill and pelagic red crab, and larval and juvenile fishes of many species are the main prey items of the blue rockfish (37–40). Algae are also a significant component of their diet during the summer months (41). FishBase estimates a trophic level of 2.8.	**Midwater**; Blue rockfish as a midwater schooling species (24, 42), and it is classified as belonging to the "subtidal and reef" assemblage based on relative abundance and co-occurrences of species within habitats (27). Blue rockfish adults show a strong affinity for kelp forests (43). Adults inhabit the midwater and surface areas around high-relief rocky reefs, within and around the kelp canopy, and around artificial reefs (44). Blue rockfish are not considered to be a migratory species (45). Primary depth range is 0-90 m and potential for adult dispersal is moderate.
6	Brown rockfish (20) 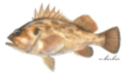	*Sebastes auriculatus*	**Tertiary carnivore;** McCain et al. (18) states that brown rockfish eat small fishes, crabs, shrimp, isopods, and polychaete worms (46, 47). An adult brown rockfish (over 30 cm) will feed on larger fish, shrimp, crabs and other crustaceans, and polychaete worms (46–52). FishBase estimates a trophic level of 4.0.	**Benthopelagic**; Brown rockfish are a bottom-associated species (24, 42), and are classified as belonging to the "kelp reef" assemblage based on relative abundance and co-occurrences of species within habitats (27). Brown rockfish are bottom dwellers, frequently living on low-profile hard bottoms (43). They aggregate near sand-rock interfaces and rocky bottoms of artificial and natural reefs over a fairly wide depth range, in eelgrass beds, near oil platforms and sewer pipes, and even around old tires (47, 53). Off California, some fish frequent sewer outfalls (46). Movements of greater than 3 km are rare for brown rockfish (54, 55), and they are said to have a strong homing tendency (47). Primary depth range is 0-130 m and potential for adult dispersal is low.
7	Brown smoothhound shark (4) 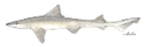	*Mustelus henlei*	**Secondary carnivore;** The brown smoothhound shark is an epibenthic predator with a diverse diet that consists of decapods, cephalopods, and small teleosts, with adults showing a preference for fish (56). Some diet studies suggest that adult brown smoothhound sharks feed overwhelmingly on teleosts, with Dendrobranchiata, Cephalopoda, Anomura and Polychaeta supplementing their diet, with limited differences across sex or size (57, 58). FishBase estimates a trophic level of 3.6.	**Benthopelagic**; Brown smoothhound sharks are a bottom-associated species (24). The brown smoothhound shark is classified as belonging to the "nearshore nocturnals" assemblage based on relative abundance and co-occurrences of species within habitats (27). This shark is thought to inhabit inshore estuarine waters from the spring through fall and move offshore in the winter months (59). Primary depth range is 0-110 m and potential for adult dispersal is moderate.
8	California Corbina (30) 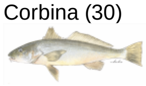	*Menticirrhus undulatus*	**Secondary carnivore;** California corbina are benthic carnivores that primarily feed on invertebrates such as bivalves, crustaceans, and polychaetes (22, 23, 60). California corbina feed by scooping sand with their mouth and filtering the contents through their gill openings. Major diet items include sand crabs (61, 62), clams, crustaceans and polychaetes (63). Shifts in the diet according to size have been noted, with California corbina less than or equal to 40 cm standard length feeding primarily on clam siphons, whereas the larger fish fed mostly on clam feet and gills (63). FishBase estimates a trophic level of 3.3.	**Benthopelagic**; Adult and juvenile California corbina are dependent upon shallow, sandy and soft bottom habitat located either along surf swept open coast, in deeper waters beyond the surf zone, or inside shallow bays (61, 62). The California corbina is classified as belonging to the "surf zone and nearshore soft bottom" assemblage based on relative abundance and co-occurrences of species within habitats (27). Primary depth range is 0-15 m and potential for adult dispersal is low.

	Species Name (sample number)	Scientific name	Diet Classification	Habitat Classification
9	California halibut (9) 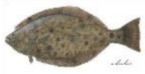	*Paralichthys californicus*	**Tertiary carnivore;** Horn and Ferry-Graham (19) classify California halibut as a tertiary carnivore among inner shelf and bay-estuarine fishes and Allen (22, 23) defined the feeding guild as bottom-living pelagivore. Adult California halibut larger than 20 cm are primarily piscivorous, with fish composing the vast majority of their prey by mass. This includes a combination of pelagic prey species such as northern anchovy, as well as benthic species such as gobies and killifish (20, 22, 64). Invertebrates that are consumed include large predatory species, such as cephalopods (64, 65). Bioaccumulation modeling parameterized California halibut diet as 98% forage fish, including both benthic and pelagic prey fish (66). FishBase estimates a trophic level of 4.5.	**Benthic;** The California halibut is a benthic species that lacks a swim bladder (65). They are generally associated with shallow soft bottom habitat such as sand or mud in estuarine, bay, and nearshore ocean waters. The California halibut is classified as belonging to the "nearshore soft bottom" assemblage based on relative abundance and co-occurrences of species within habitats (27). Primary depth range is 0-90 m and potential for adult dispersal is moderate.
10	California lizardfish (3)	*Synodus lucioceps*	**Tertiary carnivore;** Horn and Ferry-Graham (19) classify California lizardfish as a tertiary carnivore among inner and outer shelf fishes and Allen (22, 23) defined the feeding guild as bottom-living pelagivore. Allen (22) found the most important prey item to be northern anchovies and mysids in trawl surveys from the SCB coastal shelf. They are known to eat white croakers, anchovies, other fish, and squid (67). FishBase estimates a trophic level of 4.5.	**Benthic;** The California lizardfish is a benthic species that lacks a swim bladder. It is classified as belonging to the "inner mid-shelf" assemblage based on relative abundance and co-occurrences of species within habitats (27). California lizardfish are a shallow shelf species that prefers soft bottoms (22). Primary depth range is 2-230 m and potential for adult dispersal is moderate.
11	California scorpionfish (35) 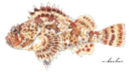	*Scorpaena guttata*	**Tertiary carnivore;** Horn and Ferry-Graham (19) classify California scorpionfish as a tertiary carnivore among outer shelf fishes and Allen (22, 23) defined the feeding guild as bottom-living ambushing benthopelagivore. Allen (22) found the most important prey items to be crabs and northern anchovies in trawl surveys from the SCB coastal shelf. Other studies have shown crabs, fish, and cephalopods to be important prey (68, 69). Looking at an analysis of 18 fish from Southern California (35), decapoda (55%), cephalopoda (28%), and fish (10%) were the most common prey items. FishBase estimates a trophic level of 3.8.	**Benthic;** The California scorpionfish is a benthic species (24) that lacks a swim bladder and is classified as belonging to the "kelp and mid-depth reef" assemblage based on relative abundance and co-occurrences of species within habitats (27). California scorpionfish are a shelf species that lives on soft and rocky bottoms as an adult (Allen 1982). Primary depth range is 0-180 m and potential for adult dispersal is low.
12	California sheephead (4) 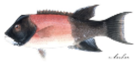	*Semicossyphus pulcher*	**Secondary carnivore;** Horn and Ferry-Graham (19) classify California sheephead as a secondary carnivore among rocky reef and kelp bed fishes. California sheephead have a varied diet. Looking at an analysis of 175 fish from Southern California (35), decapoda (21%) and amphipoda (17%) were the most abundant items by volume. Echinodermata, bivalves, and benthic worms all had between 5-10% by volume, and gastropoda, bryozoa, fish, plants, arthropods, sponges, and tunicates were all consumed in lesser amounts. FishBase estimates a trophic level of 3.6.	**Benthopelagic**; California sheephead is an epibenthic fish (24, 70). Although it can occur throughout the water column, it generally remains near the bottom (71). California sheephead is classified as a "kelp rock" or "kelp reef" assemblage species (25, 27) that occurred most abundantly in clear-water areas of high bottom relief and kelp density. Primary depth range is 0-55 m and potential for adult dispersal is low.
13	Canary rockfish (1) 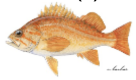	*Sebastes pinniger*	**Secondary carnivore;** McCain et al. (18) states that canary rockfish juveniles and adults primarily prey on crustaceans, primarily planktonic euphausiids and mysids, and occasionally on fish (45, 47). Canary rockfish feeding increases during the spring-summer upwelling period when euphausiids are the dominant prey and the frequency of empty stomachs is lower (72). FishBase estimates a trophic level of 3.8.	**Midwater;** Canary rockfish is a midwater schooling species (42) , and it is classified as belonging to the "kelp and mid-depth reef" assemblage based on relative abundance and co-occurrences of species within habitats (27). Canary rockfish are considered a middle shelf-mesobenthic species (73). They primarily inhabit waters 50-250 m deep (74). Canary rockfish are densely aggregating fish (47) that move into deeper water with age and also are capable of major latitudinal movements (up to 380 nautical miles) (45). Potential for adult dispersal is moderate to high.

	Species Name (sample number)	Scientific name	Diet Classification	Habitat Classification
14	Chilipepper rockfish (2) 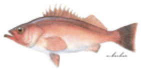	*Sebastes goodei*	**Secondary carnivore**; McCain et al. (18) states that larval and juvenile chilipepper rockfish eat all life stages of copepods and euphausiids, and are considered to be somewhat opportunistic feeders (75). In California, adults prey on large euphausiids, squid, and small fishes such as anchovy, lanternfishes, and young hake (38, 76). FishBase estimates a trophic level of 3.5.	**Midwater;** Chilipepper rockfish are a parademersal species, sometimes grouped with midwater schooling species of rockfish (42). Allen and Smith (73) define chilipepper as a middle-shelf mesobenthal to outer-shelf species and it is classified as belonging to the "deep reef and canyon" assemblage based on relative abundance and co-occurrences of species within habitats (27). Chilipepper have been taken as deep as 425 m, but nearly all in survey catches were taken between 50 and 250 m (74). In California, chilipepper are most commonly found associated with deep, high-relief rocky areas and along cliff drop-offs (76). Movements of up to 2.4 km per day have been recorded (18).
15	Chub mackerel (68) 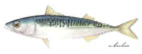	*Scomber japonicus*	**Secondary carnivore;** Horn and Ferry-Graham (19) classify chub mackerel as a secondary carnivore among epipelagic fishes. Chub mackerel feed primarily on small fishes, ichthyoplankton, squid, and crustacean zooplankton; most that are caught in the commercial fishery are less than four years (77). Looking at 440 chub mackerel from Northern California to Washington (35), 37% of the diet by weight was composed of pelagic arthropods, unidentified plant or animal material (19%), decapods (5%), and fish (3%). FishBase estimates a trophic level of 3.1.	**Pelagic;** Chub mackerel is classified as belonging to the "coastal pelagic" assemblage based on relative abundance and co-occurrences of species within habitats (27). Chub mackerel subadults and adults move northward along the coast during the summer and also exhibit inshore–offshore migration off California, moving in-shore from July through November and offshore from December through May (77, 78).
16	Copper rockfish (15) 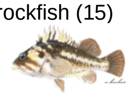	*Sebastes caurinus*	**Tertiary carnivore;** Copper rockfish are opportunistic carnivores (18). Crustaceans, followed by fish and mollusks, are the most important food groups of adult copper rockfish in terms of volume, number, and frequency of occurrence. Fishes, which include young-of-the-year rockfishes, anchovies, cusk-eels, eelpouts, and sculpins, are important forage for larger individuals (45–48, 50–52, 79). Generally, copper rockfish rely less on reef-associated food organisms as their age (size) increases (46). FishBase estimates a trophic level of 4.1.	**Benthopelagic**; Copper rockfish are a bottom-associated species (24, 42) that generally live below 55 m depth and are found on or near natural rocky reefs, boulder fields, artificial reefs, oil platforms, and rock piles. They are typically found directly on the bottom, closely associated with reefs or kelp bed areas (80–82). On high-relief rocky reefs, they maintain small home ranges (most within a 30-m^2 area), and on low-relief reefs, they have larger home ranges (80). Tagging studies indicate that copper rockfish show little movement once they have settled to the bottom. Movement of up to 1.6 km has been noted, but the majority of tagged and recaptured copper rockfish are from the locality where they were originally taken (45, 83, 84).
17	Diamond turbot (13) 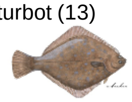	*Pleuronichthys guttulatus*	**Secondary carnivore;** Horn and Ferry-Graham (19) classify diamond turbot as a secondary carnivore among bay-estuarine fishes and Allen (22, 23) defined the feeding guild as bottom-living extracting benthivore. Juveniles feed on worms, clams and clam siphons, gastropods, ghost shrimp, amphipods, crustaceans and small fish. Adults eat sand dwelling worms, isopods, worm mollusks, clam siphons, fishes, small crustaceans such as sand crabs, and barnacles (85). FishBase estimates a trophic level of 3.3.	**Benthic;** Diamond turbot is a benthic species that lacks a swim bladder and is classified as belonging to the "nearshore bay and estuary" assemblage based on relative abundance and co-occurrences of species within habitats (27). Juveniles and adults live on the seafloor in bays, estuaries and sloughs and nearshore coastal waters on sandy or muddy bottoms. Adults are also found in sand-bottom kelp holdfast and rocky-bottom kelp-bed habitat from the surf zone to depths of 46 meters (85)
18	Fantail sole (2)	*Xystreurys liolepis*	**Secondary carnivore/ primary carnivore;** Allen (22, 23) defined the fantail sole feeding guild as bottom-living ambushing benthopelagivore. Allen (7) found the most important prey items to be crabs, gammaridean amphipods, and shrimp. Bizzaro et al. (35) contains a single individual of this species, with a diet 100% composed of amphipoda by weight. Southern California Coastal Water Research Project (66) classified fantail sole as having a benthic diet without piscivory, where	**Benthic;** Fantail sole is a benthic species that lacks a swim bladder. Fantail sole is classified as belonging to the "nearshore reef and soft bottom" assemblage based on relative abundance and co-occurrences of species within habitats (27).

	Species Name (sample number)	Scientific name	Diet Classification	Habitat Classification
			their diet consists of a mix small benthic invertebrates, such as amphipods and other crustaceans, bivalve mollusks, and polychaete worms. FishBase estimates a trophic level of 3.5.	
19	Flag rockfish (2) 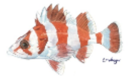	*Sebastes rubrivinctus*	**Secondary carnivore;** Flag rockfish eat mostly bottom dwellers, such as crabs, shrimp, and occasionally fish and octopus (18, 47). Allen (22, 23) defined the feeding guild as bottom-living ambushing benthopelagivore. FishBase estimates a trophic level of 3.7.	**Benthopelagic**; Flag rockfish is a bottom-associated species (42) and it is classified as belonging to the "mid depth reef and outer shelf" assemblage based on relative abundance and co-occurrences of species within habitats (27). Flag rockfish occur at depths up to 302 m (47, 86, 87), and are most common between 30 and 183 m (87). Adult flag rockfish are solitary, bottom-dwelling reef fish, although they are sometimes found in small congregations. Almost any hard bottom seems acceptable to the flag rockfish; for example, they commonly live near sewer outfalls off southern California and have been detected in submarine canyons (88).
20	Gopher rockfish (5) 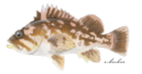	*Sebastes carnatus*	**Secondary carnivore;** Adult gopher rockfish are nighttime predators that ambush their prey (18, 25, 89). Prey items for adults include crustaceans (particularly Cancer sp. crabs, caridean shrimp, anomurans), fish (especially juvenile rockfish), and mollusks (47, 90, 45, 91). Gopher rockfish probably compete for food and space with cabezon, lingcod, greenlings, and other rockfish such as China, quillback, copper, and vermilion, based on the fact that they live in the same area. FishBase estimates a trophic level of 3.6.	**Benthopelagic**; Gopher rockfish is a bottom-associated species (24, 42), and it is classified as belonging to the "subtidal and reef" assemblage based on relative abundance and co-occurrences of species within habitats (27). Gopher rockfish generally occur in waters less than 30 m deep (92), but range from intertidal to about 86 m (82, 93). They are most commonly found between 12 and 37 m (47, 74). Gopher rockfish are shallow-water benthic rockfish that inhabit rocky reefs, kelp beds, and sandy areas near reefs (47, 94). They spend the majority of their time during the day in rocky shelters (91) and at night (and to a lesser degree during the day) perching on the bottom in the open (90). Home ranges of gopher rockfish consist of a primary shelter hole and a larger, feeding area in which they often rest in more exposed positions. The home range size increases with fish size and depth (90). Movements of more than 3 km are rare (55).
21	Gray smoothhound shark (4)	*Mustelus californicus*	**Secondary carnivore;** The gray smoothhound shark likely shares a similar diet and life history to the brown smoothhound shark, an epibenthic predator with a diverse diet that consists of decapods, cephalopods, and small teleosts. FishBase estimates a trophic level of 3.5.	**Benthopelagic**; The gray smoothhound shark is a bottom-associated species (24) classified as belonging to the "nearshore nocturnals" assemblage based on relative abundance and co-occurrences of species within habitats (27). This shark is thought to migrate from southern to central California in the summer (59). Primary depth range is 0-45 m and potential for adult dispersal is moderate.
22	Greenblotched rockfish (3) 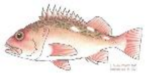	*Sebastes rosenblatti*	**Secondary carnivore;** Juvenile and adult greenblotched rockfish prey upon planktonic prey such as euphausiids and pelagic tunicates, as well as small fishes (e.g., hake, anchovies, and lanternfishes), and squid (18). Allen (22, 23) defined the feeding guild as bottom-living ambushing benthopelagivore. Off Southern California, Allen (7) reported diet differences by size class. Small fish (6.4–16.1 cm) consumed primarily shrimp, and larger fish (18.4–37.7 cm) consumed mainly fish and squid. FishBase estimates a trophic level of 3.7.	**Benthopelagic**; Greenblotched rockfish is a bottom-associated species (42) and Allen (7) grouped this rockfish species with benthic organisms (organisms without a buoyancy device). It is classified as belonging to the "deep reef and shelf" assemblage based on relative abundance and co-occurrences of species within habitats (27). Greenblotched rockfish occupy a depth range of 55-491 m (82, 86), although adults prefer depths of 61-396 m (22, 74). Adults and older juveniles are usually found near high-relief rocks, caves, and crevices, and occasionally found in mixtures of mud and rock, mud and boulders, oil platforms, and mud and cobble, with the fish lying on mud (76, 82)

	Species Name (sample number)	Scientific name	Diet Classification	Habitat Classification
23	Greenspotted rockfish (2) 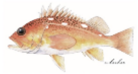	*Sebastes chlorostictus*	**Secondary carnivore;** Greenspotted rockfish are benthic feeders that prey primarily on planktonic euphausiids and pelagic tunicates, as well as small fishes (e.g., juvenile rockfishes and hake, anchovies, and lanternfishes) and squid (18, 76). Allen (22, 23) defined the feeding guild as bottom-living ambushing benthopelagivore. FishBase estimates a trophic level of 3.7.	**Benthopelagic**; Greenspotted rockfish is a bottom-associated species (42) and Allen (7) grouped this rockfish species with benthic organisms (organisms without a buoyancy device). It is classified as belonging to the “deep reef and canyon” assemblage based on relative abundance and co-occurrences of species within habitats (27). Greenspotted rockfish are common, benthic inhabitants in waters 90–363 m deep (82, 86). Adult greenspotted rockfish prefer waters 49-201 m deep (74). Greenspotted rockfish spend most of their time on or near the bottom, often in caves and crevices. Adult greenspotted rockfish are mostly caught over high-relief rocky reefs (76), but they are also common on soft bottoms (94), such as sand or mud (55). They are frequently observed on mud near rock outcrops, and less frequently near oil platforms (82). Greenspotted rockfish do not undergo extensive migrations or movements as they are sedentary creatures which rarely venture a few feet above the rocks they inhabit (47).
24	Halfmoon (3) 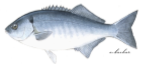	*Medialuna californiensis*	**Herbivore;** Horn and Ferry-Graham (19) classify halfmoon as a herbivore among rocky reef and kelp bed fishes. An analysis of 15 fish from Southern California (35) showed 64% of the diet by volume was composed of plant material, followed by tunicates (13%) and Cnidarian (6%). FishBase estimates a trophic level of 2.7.	**Midwater;** Halfmoon are a water column species that utilizes the kelp canopy (24) and their habitat is defined as kelp reef (27). Halfmoon are commonly found at the outer edges of kelp beds, over shallow rocky areas, and in kelp beds (95). They have been observed as deep as 40 meters but are most commonly taken by anglers from waters from 2 to 20 meters deep (95).
25	Hornyhead turbot (48)	*Pleuronichthys verticalis*	**Secondary carnivore;** Horn and Ferry-Graham (19) classify hornyhead turbot as a secondary carnivore among inner and outer shelf fishes and Allen (22, 23) defined the feeding guild as bottom-living extracting benthivore. Allen (7) found the most important prey were polychaete worms, with bivalves and other benthic organisms also found. FishBase estimates a trophic level of 3.1.	**Benthic;** Hornyhead turbot is a benthic species that lacks a swim bladder. Hornyhead turbot is classified as belonging to the “inner mid-shelf” assemblage based on relative abundance and co-occurrences of species within habitats (27).
26	Jacksmelt (3) 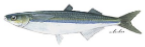	*Atherinopsis californiensis*	**Secondary carnivore;** FishBase estimates a trophic level of 3.1.	**Midwater;** Jacksmelt is a water-column species (24) classified as belonging to the “nearshore generalist” assemblage based on relative abundance and co-occurrences of species within habitats (27).
27	Kelp bass (100) 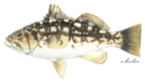	*Paralabrax clathratus*	**Secondary carnivore;** Horn and Ferry-Graham (19) classify kelp bass as a secondary carnivore among rocky reef and kelp bed fishes. The kelp bass is a major fish predator around rocky reefs and kelp beds, with young bass feeding on small crabs, copepods, and plankton before assuming a generalized carnivore diet of small fishes, including anchovies, sardines, surfperches, and queenfish, and a variety of macroinvertebrates. Looking at an analysis of 111 fish from Southern California (35), 30% of the diet by volume was composed of decapoda, followed by amphipoda (26%), benthic arthropods (20%), fish (12%), and to a lesser extent pelagic arthropods (4%), and benthic worms (4%). FishBase estimates a trophic level of 3.9.	**Midwater;** Kelp bass is primarily a water column species (24) that also utilizes bottom habitat. Kelp bass primarily reside in nearshore habitats, including kelp forests, bays, and estuaries and occur throughout the water column (25, 71). Kelp bass is classified as belonging to the “shallow rock sand” assemblage based on relative abundance and co-occurrences of species within habitats (27). Primary depth range is 0-23 m and potential for adult dispersal is moderate.
28	Kelp rockfish (1)	*Sebastes atrovirens*	**Secondary carnivore**; Kelp rockfish are carnivorous and eat a variety of prey, most of which are free-swimming (18, 47). They are most active at night and will	**Midwater;** Kelp rockfish is a midwater schooling species (24, 42), and it is classified as belonging to the “kelp reef” assemblage based on relative

	Species Name (sample number)	Scientific name	Diet Classification	Habitat Classification
	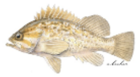		sometimes chase food slightly away from the plant habitat (47). Older kelp rockfish prey primarily on benthic invertebrates and small fishes that colonize the substrata; they ambush these prey a short distance from kelp fronds (96, 97). An index of relative importance shows that their diet is dominated by caridean shrimp and amphipods; tunicates, cephalopods, and gastropods are also important (96). Adult kelp rockfish also commonly prey on juvenile rockfishes (98). FishBase estimates a trophic level of 3.4.	abundance and co-occurrences of species within habitats (27). Kelp rockfish inhabit shallow waters. Most live at depths of 18-24 m (82) although they occur from 3 to 58 m (82, 94). As adults, kelp rockfish are primarily residential (55) in kelp forests and are considered parademersal (98). Kelp rockfish is a shallow-water species, which is less likely to undertake movements than species inhabiting deeper waters (55). They do not make extensive seasonal migrations (55). However, during winter storms they may migrate into slightly deeper water or retire to rock caves, otherwise they rarely move from place to place (47).
29	Leopard shark (4) 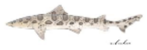	*Triakis semifasciata*	**Tertiary carnivore**; The leopard shark utilizes several major food sources without depending upon one (99), and feeding habits are dependent upon the size of the shark (18). Juveniles and adults are carnivorous, opportunistic, benthic and littoral feeders. Leopard sharks 90–120 cm in length feed mostly on echiuroid worms. Sharks 120–130 cm feed on crabs, clam siphons, fishes, fish eggs, and echiuroid worms (100). Fishes make up the greatest portion of food eaten by 130–140 cm long sharks (99). Leopard sharks also prey upon polychaete worms and octopi and feed rapidly on the eggs of herring, topsmelt, jacksmelt, and midshipmen when available (47, 101). Presence of mud-burrowing prey in their diet signifies that the leopard shark is feeding very close to or in the mud (101). The leopard shark must display a sucking or digging behavior to remove clam siphons and echiuroid worms from the mud (99, 102). FishBase estimates a trophic level of 3.7.	**Benthopelagic**; Leopard shark is a bottom-associated species (24). Leopard shark is classified as belonging to the “nearshore nocturnals” assemblage based on relative abundance and co-occurrences of species within habitats (27). A coastal species, the leopard shark is most abundant in northern California bays and estuaries and along southern California beaches (100). Although they are common in enclosed, muddy bays, other habitats of the leopard shark are flat, sandy areas, mud flats, sandy and muddy bottoms strewn with rocks near rocky reefs, and kelp beds (47, 94, 101, 103–105). Leopard sharks are most common on or near the bottom in waters less than 20 m deep, but have been caught as deep as 91 m (104, 106).
30	Longfin sanddab (3)	*Citharichthys xanthostigma*	**Secondary carnivore;** Horn and Ferry-Graham (19) classify longfin sanddab as a secondary carnivore among outer shelf fishes and Allen (22, 23) defined the feeding guild as bottom-living pelagobenthivores. Diet is likely similar to Pacific sanddab and speckled sanddab, consisting mostly of polychaetes, epibenthic crustacea, and planktonic crustacea (22). FishBase estimates a trophic level of 3.6.	**Benthic;** Longfin sanddab is a benthic species that lacks a swim bladder and is classified as belonging to the “inner mid-shelf” assemblage based on relative abundance and co-occurrences of species within habitats (27).
31	Market squid (26) 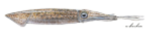	*Doryteuthis opalescens*	**Primary carnivore**; California market squid forages primarily on crustacean zooplankton (107) and has a relatively short life span of approximately six to nine months (108). Market squid have a mixed, size dependent diet. Looking at an analysis of 119 squid from Northern California, Oregon, and Washington (35), 59% of the diet by weight was composed of decapoda, followed by unidentified animal or invertebrate material (20%), fish (16%), and pelagic zooplankton (4%). FishBase estimates a trophic level of 2.5.	**Pelagic;** Market squid is classified as belonging to the “coastal pelagic” assemblage based on relative abundance and co-occurrences of species within habitats (27).
32	Northern anchovy (24) 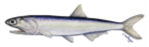	*Engraulis mordax*	**Primary carnivore;** Horn and Ferry-Graham (19) classify northern anchovy as a primary carnivore among shelf and epipelagic fishes and Allen (22, 23) defined the feeding guild as water-column schooling pelagivores. Northern anchovy generally live to approximately three to four years and feed by filtering or engulfing crustacean zooplankton and ichthyoplankton (109). Northern anchovies have a pelagic dependent diet. Looking at an analysis of 222 fish from Northern California, Oregon, and Washington (35), 33% of the diet	**Pelagic;** Northern anchovy is classified as belonging to the “coastal pelagic” assemblage based on relative abundance and co-occurrences of species within habitats (27). The central subpopulation of Northern anchovy in the SCB is known to migrate southward and offshore for winter spawning (78).

	Species Name (sample number)	Scientific name	Diet Classification	Habitat Classification
			by weight was composed of unidentified invertebrates or animals, followed by pelagic arthropods (26%), plant material (26%), decapods (12%), and fish (1%). FishBase estimates a trophic level of 3.1.	
33	Ocean whitefish (2) 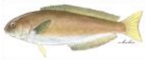	*Caulolatilus princeps*	**Secondary carnivore**; Ocean whitefish will feed on a large variety of benthic prey items including crustaceans (shrimp, crabs, and krill), small octopus, squid and small fish (110). FishBase estimates a trophic level of 4.0.	**Benthopelagic;** Ocean whitefish is a bottom-associated species (24). Ocean whitefish is classified as belonging to the "kelp reef" assemblage based on relative abundance and co-occurrences of species within habitats (27). Juveniles are found in low reef habitats and sandy bottoms (47). However, adults split their time between day and night habitats at depths of 18 to 68 m; during the day they are commonly found in deep sand habitats and during the night in shallow high-relief structure or kelp beds (111).
34	Olive rockfish (1) 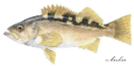	*Sebastes serranoides*	**Secondary carnivore;** Horn and Ferry-Graham (19) classify olive rockfish as a secondary carnivore among rocky reef and kelp bed fishes. Adults and subadults rockfish feed primarily on midwater organisms rather than on substrata-orientated prey (18). They also feed more on moving prey and so may forage more widely than other species of rockfish (112). Major prey of the olive rockfish include fishes (particularly juvenile rockfishes), octopi, squid, and planktonic organisms, such as copepods and crab larvae (47)(Love 1996), although polychaetes are sometimes consumed (39, 45, 47, 49, 52, 69, 98, 113–115). Olive rockfish prefer fish prey over plankton, and the fish consumed include juvenile blacksmith, anchovy, pipefish, blue rockfish, olive rockfish, adult topsmelt, and anchovy (39). FishBase estimates a trophic level of 3.9.	**Midwater;** Kelp rockfish is a water-column species (24, 42), and it is classified as belonging to the "subtidal and reef" assemblage based on relative abundance and co-occurrences of species within habitats (27). Olive rockfish occur from surface/intertidal waters to 174 m deep (47, 94, 112). Most commonly they occur in waters less than 30 m (94). Adult olive rockfish are a midwater fish, almost always living over hard, high relief (such as reefs, wrecks, oil platforms, or pipes; Love 1996). They are mostly a sedentary fish (Love 1996) and tagging studies show that they tend to spend their entire life near the same reef (115).
35	Opaleye (20) 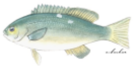	*Girella nigricans*	**Herbivore;** Horn and Ferry-Graham (19) classify opaleye as a herbivore among rocky reef and kelp bed fishes. Opaleye are primarily herbivorous (116). Looking at an analysis of 13 opaleye from Southern California (35), 68% of the diet by volume was composed of plant material, followed by Cnidarina (14%) and Amphipoda (11%). FishBase estimates a trophic level of 2.2.	**Benthopelagic**; Opaleye generally roam the kelp-bed floors and sometimes the canopy (71). Stephens et al. (24) classifies them as a water-column species, but also states that they are an epibenthic fish typically associated with rocky bottoms in kelp forests. Opaleye is classified as belonging to the "kelp reef" assemblage based on relative abundance and co-occurrences of species within habitats (27). Ebeling et al. (25) classified opaleye as a kelp-rock assemblage species that occurred most abundantly in clear-water areas of high bottom relief and kelp density. Primary depth range is 0-30 m and potential for adult dispersal is moderate.
36	Pacific barracuda (3) 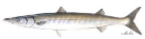	*Sphyraena argentea*	**Tertiary carnivore**; Barracuda feed upon a variety of smaller fish species including anchovies, sardines, young Pacific mackerel, young jack mackerel, and California grunion (117). FishBase estimates a trophic level of 4.5.	**Pelagic;** Pacific barracuda is classified as belonging to the "coastal pelagic" based on relative abundance and co-occurrences of species within habitats (27). Pacific barracuda are migratory, and the presence of Pacific barracuda off the coast of California is highly variable and dependent on environmental conditions (117).
37	Pacific sardine (35) 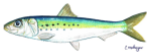	*Sardinops sagax*	**Primary carnivore;** Horn and Ferry-Graham (2006) classify pacific sardine as a primary carnivore among epipelagic fishes. Most Pacific sardines live to three to seven years and feed by filtering crustacean zooplankton and ichthyoplankton (107). Looking at an analysis of 181 Pacific sardines from Northern California to Washington	**Pelagic;** Pacific sardine is classified as belonging to the "coastal pelagic" based on relative abundance and co-occurrences of species within habitats (27). Although affected by oceanographic factors, Pacific sardine migrations typically are northward during the

	Species Name (sample number)	Scientific name	Diet Classification	Habitat Classification
			(35), 61% of the diet by weight was composed of plant material (36%), pelagic arthropods (28%), unidentified plant or animal material (18%), tunicates (7%), and decapoda (6%). FishBase estimates a trophic level of 2.8.	early summer and southward beginning in the fall (78, 107).
38	Pile surfperch (5) 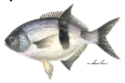	*Phanerodon vacca*	**Primary carnivore;** Horn and Ferry-Graham (19) classify pile surfperch as a secondary carnivore among rocky reef and kelp bed fishes. Pile surfperch primarily rely on benthic material. Looking at an analysis of 15 pile surfperch from Southern California (35), 61% of the diet by volume was composed of gastropoda, followed by bivalves (28.4%). FishBase estimates a trophic level of 3.0.	**Benthopelagic**; Pile surfperch is a bottom-associated species (24). They are a schooling species and can be found throughout the water column (25, 71). Pile surfperch is classified as belonging to the "shallow rock sand" based on relative abundance and co-occurrences of species within habitats (27). Primary depth range is 0-45 m and potential for adult dispersal is moderate.
39	Queenfish (17) 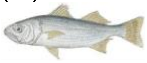	*Seriphus politus*	**Secondary carnivore;** Horn and Ferry-Graham (19) classify queenfish as a secondary carnivore among inner shelf fishes. Allen (22, 23) defined the feeding guild as water-column schooling pelagivores, although based on a collection of previous studies it appears that queenfish have a mixed benthic and pelagic diet with piscivory. (118) found that approximately 90% of queenfish prey were northern anchovy. In contrast, (119) found that mysids were the predominant prey item (45%), followed by amphipods (22%), annelid worms (22%), with very small contributions of shrimp, isopods, and fish. Allen (22) found the most important prey to be mysids, followed by (mostly larval) reptantian decapods and copepods. Looking at an analysis of 34 fish from Southern California (35), 48% of the diet by volume was composed of benthic arthropods, followed by worms (20%), amphipoda (19%), decapoda (11%), and fish (1%). Southern California Coastal Water Research Project (66) used food web model parameters for queenfish as follow: pelagic forage fish (48%), mysids (24%), amphipod crustaceans (12%), large and small polychaetes (5% and 6%,respectively), crangonid shrimp (3%), and cumacean crustaceans (2%). FishBase estimates a trophic level of 3.7.	**Midwater;** Queenfish is a water-column species (24) classified as belonging to the "nearshore soft bottom" based on relative abundance and co-occurrences of species within habitats (27). Queenfish has an association with soft bottom sediments. Primary depth range is 0-55 m and potential for adult dispersal is moderate.
40	Quillback rockfish (1) 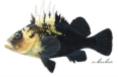	*Sebastes maliger*	**Secondary carnivore**; McCain et al. (18) states that quillback rockfish consume a wide range of prey taxa and are more dietary generalists than other rockfish species (120). As adults their habit is more benthic, and they are known to feed on a variety of prey such as crustaceans, small fish including rockfishes and flatfishes, bivalves, polychaetes, and fish eggs such as from lingcod (47, 50, 120–122). FishBase estimates a trophic level of 3.8.	**Benthopelagic**; Quillback rockfish are a common, shallow-water benthic species. They are taken from subtidal depths to 275 m (38, 47) but they occur mainly from 9 to 147 m (74). Quillback rockfish are solitary reef-dwellers, living close to or on the bottom (47, 120, 123). Occasionally they will rise up 9–12 m in the water column (47). Tagging studies in central California and Washington have shown quillback to be residential (no movement other than diurnal) or to show movement of less than 9.6 km (45, 124).
41	Rainbow surfperch (9) 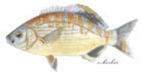	*Hypsurus caryi*	**Primary carnivore;** Generally, these fish consume small mollusks and polychaetes. Looking at an analysis of 9 fish from central California to Washington (35), 60% of the diet by volume was composed of amphipods, followed by pelagic arthropods (21%). FishBase estimates a trophic level of 3.3.	**Benthopelagic**; Rainbow surfperch is a bottom-associated species (24) classified as belonging to the "subtidal and reef" based on relative abundance and co-occurrences of species within habitats (27). Ebeling et al. (25) classified rainbow surfperch as an inner-marginal assemblage species that occurred shoreward at shallow depth where seagrass was plentiful.

	Species Name (sample number)	Scientific name	Diet Classification	Habitat Classification
42	Rosethorn rockfish (3) 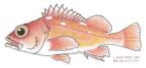	*Sebastes helvomaculatus*	**Secondary carnivore;** Off central California, principal prey items are euphausiids and other crustaceans (18). FishBase estimates a trophic level of 3.7.	**Benthopelagic**; Rosethorn rockfish is a bottom-associated species (42), and it is classified as belonging to the “deep reef and canyon” based on relative abundance and co-occurrences of species within habitats (27). Rosethorn rockfish occur in water 25-549 m deep (38, 82, 125–127), although most occur from 100 to 350 m (73), and are generally categorized with other deep-water rockfishes (126, 127). Adults are generally found in muddy areas adjacent to boulders, cobble, or rock; occasionally they are found in rocky areas without mud, and in association with sea lilies (82).
43	Shiner surfperch (30) 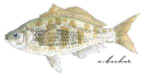	*Cymatogaster aggregata*	**Primary carnivore;** Horn and Ferry-Graham (2006) classify shiner surfperch as a secondary carnivore among inner shelf fishes and a primary carnivore among bay-estuarine fishes. Allen (22, 23) defined the feeding guild as water-column pelagobenthivores. Shiner surfperch are generally epibenthic feeders, primarily feeding off the sediment surface or on epifauna of hard structures. (128) reported that for Anaheim Bay shiner perch, the primary food source was zooplankton and benthic organisms, including bivalves, gastropods, polychaetes, tunicates, and fish eggs. Similarly, Allen (22) found the most important diet items to be calanoid copepods and chaetognaths. Southern California Coastal Water Research Project (66) used food web model parameters for shiner surfperch as follows: sediments (5%), benthic polychaete worms (20%), amphipod crustaceans (20%), and cumacean crustaceans (20), benthopelagic mysids (15%), and pelagic phytoplankton (10%) and zooplankton (10%). Looking at an analysis of 84 fish from Southern California (35), 50% of the diet by volume was composed of pelagic arthropods, followed by benthic amphipods (31%) and benthic arthropods (7%). FishBase estimates a trophic level of 3.0.	**Midwater;** Shiner surfperch is a water-column species (24) classified as belonging to the “nearshore generalist” assemblage based on relative abundance and co-occurrences of species within habitats (27). They are found in shallow habitats during the spring to fall months, typically in estuaries and bays, associated with kelp canopy, subtidally near beaches, and in proximity to man-made structures such as piers and pilings (38, 128–131). In California, eelgrass habitat provides important foraging, spawning, and nursery habitat for Shiner surfperch particularly in bays and estuaries (128, 130). In winter, Shiner Perch transition to deeper waters and forage on benthic invertebrates and fish eggs when plankton become less abundant in shallow waters (128). Juveniles and adults primarily school in nearshore waters at 1.5 to 15 m depth, although they can be found in depths up to 29 m (130).
44	Shovelnose guitarfish (6) 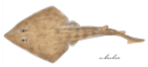	*Rhinobatos productus*	**Secondary carnivore;** Horn and Ferry-Graham (2006) classify shovelnose guitarfish as a secondary carnivore among inner shelf fishes. Shovelnose guitarfish primarily rely on benthic material. Looking at an analysis of 5 fish from Southern California (35), 61% of the diet by volume was composed of bivalves, followed by decapoda (16%), amphipoda (14%), and benthic worms (8%). FishBase estimates a trophic level of 3.6.	**Benthic;** Shovelnose guitarfish is a bottom-associated species (24). Shovelnose guitarfish is classified as belonging to the “nearshore soft bottom” assemblage based on relative abundance and co-occurrences of species within habitats (27). Primary depth range is 0-15 m and potential for adult dispersal is moderate.
45	Slough anchovy (4)	*Anchoa delicatissima*	**Primary carnivore;** Horn and Ferry-Graham (2006) classify slough anchovy as a primary carnivore among bay-estuarine fishes. FishBase estimates a trophic level of 3.4.	**Pelagic;** Slough anchovy is classified as belonging to the “bay-estuary” based on relative abundance and co-occurrences of species within habitats (27).
46	Speckled rockfish (9) 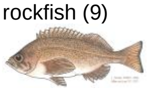	*Sebastes ovalis*	**Secondary carnivore;** Speckled rockfish feed primarily on plankton, although they will occasionally eat small fish (47). FishBase estimates a trophic level of 3.8.	**Midwater;** Speckled rockfish are a water-column species (42) that occur in water 18-366 m deep and are most common between 76 and 152 m (47, 86). They are an aggregating species, and likely move from reef to reef (47)
47	Speckled sanddab (3)	*Citharichthys stigmaeus*	**Secondary carnivore;** Horn and Ferry-Graham (2006) classify speckled sanddab as a secondary carnivore among inner shelf fishes and Allen (22, 23) classified it as a bottom-living pelagobenthivore. Allen (22) found that in the Southern California Bight, the most important prey	**Benthic;** Speckled sanddab is a benthic species that lacks a swim bladder and is classified as belonging to the “nearshore algal bed and soft bottom” assemblage based on relative abundance and co-occurrences of species within habitats (27).

	Species Name (sample number)	Scientific name	Diet Classification	Habitat Classification
			items were mysids, gammaridean amphipods, reptantian decapods, and echiurids. Polychaetes and small fish were also found occasionally. Looking at an analysis of 22 fish from Southern California and Central/Northern California, Oregon, Washington (35), 56% of the diet by volume was composed of amphipoda, followed by benthic worms (21%) and benthic arthropods (13%). FishBase estimates a trophic level of 3.4.	
48	Spiny dogfish (1) 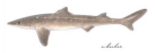	*Squalus acanthias*	**Tertiary carnivore;** Allen (22, 23) defined the spiny dogfish feeding guild as water-column cruising pelagobenthivores. They are carnivorous scavengers and McCain et al. (2019) states that they are an opportunistic feeder, taking whatever is available. Their diet consists primarily of fish, especially sandlance, herring, smelts, cods, capelin, hake, and ratfish, and of invertebrates, particularly shrimp, crabs, worms, krill, squid, octopus, jellyfish, and sea cucumbers (132, 133). Fish become a more important dietary source as the dogfish grow larger (103, 134, 135). Most of the diet of juveniles consists of pelagic prey, generally small invertebrates, whereas the adults prey largely on benthic organisms (133). An analysis of 120 fish from Northern California, Oregon, and Washington (35), shows 40% of the diet by weight was composed of unidentified animal or invertebrate material and 37% by fish. FishBase estimates a trophic level of 4.3.	**Midwater**; Spiny dogfish is classified as belonging to the "deep shelf, bank, and slope" based on relative abundance and co-occurrences of species within habitats (27). Spiny dogfish is an inner shelf-mesobenthal species with a depth range of 0–1236 m, but typically inhabiting waters less than 350 m deep (73). In southern California, Spiny dogfish are often found in close association with white croaker (103, 135). Spiny dogfish often migrate long distances in large schools, and feed avidly on their journeys (132). Seasonal migrations are taken so as to stay in the preferred temperature range (135).
49	Spotfin croaker (6) 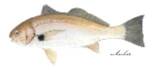	*Roncador stearnsii*	**Secondary carnivore;** Southern California Coastal Water Research Project (66) classified spotfin croaker as having a benthic diet without piscivory, where their diet consists of a mix small benthic invertebrates, such as amphipods and other crustaceans, bivalve mollusks, and polychaete worms. FishBase estimates a trophic level of 3.3.	**Benthopelagic**; Spotfin croaker is classified as belonging to the "surf zone and nearshore soft bottom" assemblage based on relative abundance and co-occurrences of species within habitats (27).
50	Spotted sand bass (35) 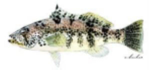	*Paralabrax maculatofasciatus*	**Tertiary carnivore;** Horn and Ferry-Graham (2006) classify spotted sand bass as a tertiary carnivore among bay-estuarine fishes. Two studies were available to develop quantitative dietary composition for spotted sand bass (136, 137). Both studies reported decapod crabs as the second most important prey type. Allen et al. (136) reported mollusks to be the primary prey type, while Mendoza-Carranza (137) indicated fishes as the most important prey. Southern California Coastal Water Research Project (66) set bioaccumulation model input parameters to represent benthic and pelagic fishes (35%), crabs (35%) and mollusks (28%) as the major prey items, with phytoplankton and amphipods each included as 1% of total diet. FishBase estimates a trophic level of 4.2.	**Benthopelagic**; Spotted sand bass is classified as belonging to the "nearshore bay and estuary" assemblage based on relative abundance and co-occurrences of species within habitats (27). Spotted sand bass juveniles and adults are dependent upon warm, shallow water embayments such as harbors, estuaries, bays, and lagoons. Some adults can be found in more exposed habitat just outside of these harbors and bays, but most occur inside. Eelgrass beds within these embayments serve as nursery areas, and adult spotted sand bass are also common in these eelgrass beds (138). Primary depth range is 0-60 m and potential for adult dispersal is low.
51	Spotted turbot (8)	*Pleuronichthys ritteri*	**Secondary carnivore;** Horn and Ferry-Graham (2006) classify spotted turbot as a secondary carnivore among inner shelf fishes. Allen (22, 23) classifies it as a bottom-living extracting benthivore. FishBase estimates a trophic level of 3.2.	**Benthic;** Spotted turbot is a benthic species that lacks a swim bladder and is classified as belonging to the "nearshore soft bottom" assemblage based on relative abundance and co-occurrences of species within habitats (27).
52	Squarespot rockfish (4) 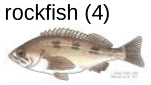	*Sebastes hopkinsi*	**Primary carnivore;** McCain et al. (2019) states that squarespot rockfish feed entirely on plankton, primarily copepods, krill, and crab larvae (47). FishBase estimates a trophic level of 3.6.	**Midwater;** Squarespot rockfish are a water-column species (24, 42) and are classified as belonging to the "mid-depth reef" based on relative abundance and co-occurrences of species within habitats (27).

	Species Name (sample number)	Scientific name	Diet Classification	Habitat Classification
				Squarespot rockfish occur in water 18–224 m deep and are most common between 30 and 150 m (82, 86). Squarespot rockfish are found over high rocky reefs and in areas with cobble (47, 82, 139). Squarespot rockfish tend to form schools, often consisting of hundreds to thousands individuals (47).
53	Starry rockfish (9) 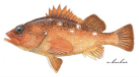	*Sebastes constellatus*	**Secondary carnivore;** McCain et al. (2019) states that starry rockfish diet consists of small fishes, crabs, shrimp, and other small invertebrates (47). FishBase estimates a trophic level of 3.6.	**Benthopelagic**; Starry rockfish is a bottom-associated species (42), and it is classified as belonging to the "mid-depth reef" assemblage based on relative abundance and co-occurrences of species within habitats (27). Starry rockfish have an overall depth range of 24–274 m (74), but are most commonly found at depths of 60–150 m off of southern California (82). Starry rockfish are generally solitary. They live directly on the ocean bottom and rarely move more than 0.5 m above the reef (18). Starry rockfish are exclusively found over hard bottoms, usually around large rocks, boulders, and occasionally over cobble or wrecks. It is unlikely that they move from reef to reef (3).
54	Striped mullet (3)	*Mugil cephalus*	**Herbivore;** Horn and Ferry-Graham (2006) classify striped mullet as a combination herbivore and detritivore among bay-estuarine fishes. Striped mullet are adapted to consume plant material, with most dietary studies indicating sizable contributions of plants and algae, as well as detritus (i.e., sediments). They are unusual among marine fish in California in that sediments and plant material often constitute the majority of their diet (140–142). Southern California Coastal Water Research Project (66) set bioaccumulation model input parameters with 75% of the diet composed of sediments and plant material (30% sediments, 35% benthic macrophytes, and 10% phytoplankton), zooplankton (10%), and benthic invertebrates (5% mollusks, 5% amphipods, and 5% polychaetes). FishBase estimates a trophic level of 2.5.	**Midwater;** Striped mullet is classified as belonging to the "nearshore bay and estuary" assemblage based on relative abundance and co-occurrences of species within habitats (27).
55	Topsmelt (10) 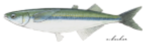	*Atherinops affinis*	**Primary carnivore;** Horn and Ferry-Graham (2006) classify topsmelt as a herbivore among bay-estuarine fishes. Generally, topsmelt diets include benthic and pelagic invertebrates, benthic algae, and phytoplankton, although studies offer conflicting evidence as to the extent of herbivory. Some find substantial contributions of benthic herbivory (142) while others find diets primarily composed of benthic and pelagic invertebrates (143, 144). An analysis of 26 southern California topsmelt (35) found that pelagic arthropods dominated the diet (82%) followed to a lesser extent by benthic arthropods (6%) and fish (4%). Southern California Coastal Water Research Project (66) set bioaccumulation model input parameters as follows: phytoplankton (20%), submerged plants (20%), benthic amphipods (40%), and minor contributions from zooplankton (8%), sediments (5%), mysids (5%), polychaetes (1%), and cumacean crustaceans (1%). FishBase estimates a trophic level of 2.8.	**Midwater;** Topsmelt is a water-column species (24) classified as belonging to the "nearshore generalist" assemblage based on relative abundance and co-occurrences of species within habitats (27). They inhabit surface waters and are rarely found below 15 feet (71).
56	Vermillion rockfish (68) 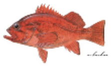	*Sebastes miniatus*	**Secondary carnivore;** Allen (22, 23) defined the vermillion rockfish feeding guild as water-column, bottom-refuge visual pelagivores. McCain et al. (18) states that vermilion rockfish prey on other fishes (anchovies, lanternfishes, small rockfishes), octopi,	**Benthopelagic**; Vermillion rockfish are a bottom-associated species (24, 42), and it is classified as belonging to the "kelp and mid-depth reef" assemblage based on relative abundance and co-occurrences of species within habitats (27). Adults

	Species Name (sample number)	Scientific name	Diet Classification	Habitat Classification
			squids, and krill (47). FishBase estimates a trophic level of 3.8.	occur at depths up to 436 m, and commonly occur at depths of 50-150 m (82, 145). Adults occur mostly on or near the bottom in areas with high-relief rocky reefs, rarely rising more than 3 m above the bottom, and they are occasionally associated with oil platforms and kelp beds (47, 82, 146). Results of tagging studies conducted off central California suggested that this species has strong site fidelity and moves very little from its primary habitat type (45).
57	Walleye surfperch (5) 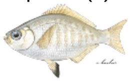	*Hyperprosopon argenteum*	**Secondary carnivore;** Horn and Ferry-Graham (2006) classify walleye surfperch as a secondary carnivore among inner shelf fishes and Allen (22, 23) defined the feeding guild as water-column schooling pelagivore. Walleye surfperch primarily rely on benthic material. Allen (22) found that mysids were by far the most common, abundant, and volumetrically important prey. Other studies found gammaridean amphipods, cumacean crustaceans, isopods, small fish, and shrimps to be important prey items (32, 69, 147). Looking at an analysis of 51 fish from Southern California (35), 55% of the diet by volume was composed of gammaridean amphipods, followed by benthic arthropods (34%). FishBase estimates a trophic level of 3.5.	**Midwater;** Walleye surfperch is a water-column species (24) classified as belonging to the “surf zone and nearshore soft bottom” assemblage based on relative abundance and co-occurrences of species within habitats (27). It occurs most abundantly in clear-water areas of high bottom relief and kelp density (25). During the day it aggregates in dense, inactive schools in shallow water along sandy beaches, sand-rock areas, or in the surf (89, 147). At night the schools disperse and the fish swim individually or in small groups in the water column (147). Primary depth range is 0-18 m and potential for adult dispersal is moderate.
58	White croaker (138) 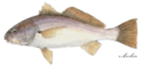	*Genyonemus lineatus*	**Secondary carnivore;** Horn and Ferry-Graham (2006) classify white croaker as a secondary carnivore among inner shelf fishes and Allen (22, 23) defined the feeding guild as water-column cruising nocturnal benthopelagivore. White croaker is a bottom feeder, predominantly consuming benthic invertebrates and fishes such as amphipods, copepods, and polychaetes (148). Allen (22) found that the most common food items were polychaetes, crabs, amphipods, and chaetognaths. Previously validated case studies have used food web model parameters for white croaker as follows: polychaetes (40%), amphipod crustaceans (20%), and cumacean crustaceans (20%), benthopelagic mysids (10%) and crangon shrimp (5%) (66, 149). FishBase estimates a trophic level of 3.4.	**Benthopelagic**; White croaker is classified as belonging to the “nearshore soft bottom and inner-shelf” assemblage based on relative abundance and co-occurrences of species within habitats (27). It is a shallow shelf species that prefers soft bottoms (22). Adult white croaker are found in cloudy nearshore waters and within bays over muddy substrate. Primary depth range is 0-130 m and potential for adult dispersal is low. Adults are found near the benthos, but occasionally they will rise to the surface when chasing prey (131). In southern California, white croaker congregate near the Los Angeles County sewage outfall site at White Point, Palos Verdes and other polluted areas such as the Los Angeles/Long Beach Harbor (150).
59	White seaperch (10)	*Phanerodon furcatus*	**Primary carnivore;** Horn and Ferry-Graham (2006) classify white seaperch as a secondary carnivore among inner shelf fishes. Allen (22, 23) classified it as a water-column cruising diurnal benthopelagivore and found the most important prey were gammaridean amphipods and reptantian decapods, although polychaetes also occurred frequently. Looking at an analysis of 9 fish from Southern California (35), 38% of the diet by volume was composed of amphipods, followed by bivalves (30%), plant material (14%), and benthic worms (10%). FishBase estimates a trophic level of 3.4.	**Midwater**; White seaperch is a bottom-associated species (24) that also utilizes the water-column. It swims in loose schools or aggregations; during the day it is generally found in midwater or just above the bottom, and it is found in midwater or on the bottom at night (151, 152). Ebeling et al. (25) classified white surfperch as a commuter assemblage species that occurred most abundantly in clear-water areas of high bottom relief and kelp density. White seaperch is classified as belonging to the “shallow rock sand” assemblage based on relative abundance and co-occurrences of species within habitats (27). The white seaperch is found for an sandy and rocky areas, but prefers sandy areas to reef areas (33, 34). Primary depth range is 0-43 m and potential for adult dispersal is moderate.
60	Yellowfin croaker (38)	*Umbrina roncador*	**Secondary carnivore;** Horn and Ferry-Graham (2006) classify yellowfin croaker as a secondary carnivore among bay-estuarine fishes. Southern California Coastal Water Research Project (66) classified them as having a	**Benthopelagic**; Yellowfin croaker is classified as belonging to the “nearshore nocturnals” assemblage based on relative abundance and co-occurrences of species within habitats (27). Yellowfin croaker

	Species Name (sample number)	Scientific name	Diet Classification	Habitat Classification
	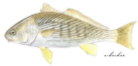		benthic diet with piscivory, where their diet consists of a mix of benthic invertebrates and forage fish. FishBase estimates a trophic level of 3.5.	generally reside over shallow, sandy, or muddy bottom habitat that occurs on the open coast or within bays and lagoons (61), though they may also venture into reef habitat (153). Primary depth range is 0-45 m and potential for adult dispersal is low.
61	Yellowtail rockfish (4) 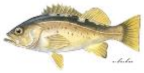	*Sebastes flavidus*	**Secondary carnivore;** Yellowtail rockfish feed mainly on pelagic animals, but are opportunistic, occasionally eating benthic animals as well (18, 154). Large juveniles and adults eat fish (e.g., small hake, Pacific herring, smelt, anchovies, lanternfishes, and others) along with squid, krill, and other planktonic organisms (e.g., euphausiids, mysids, salps, and pyrosomes) (47, 155, 156). FishBase estimates a trophic level of 3.5.	**Midwater;** Yellowtail rockfish is a water-column species (42), and it is classified as belonging to the "kelp and mid-depth reef" assemblage based on relative abundance and co-occurrences of species within habitats (27). Yellowtail rockfish is most abundant over the middle shelf between 90 and 180 m depth (82, 156–159). As adults, yellowtail are considered semi-pelagic (160, 161) or pelagic, because they range over wider areas than benthic rockfish and can make long distance movements (161). Movements of up to 158 km in tag-and-release studies have been reported (45). Adult yellowtail rockfish show strong site fidelity and homing abilities (157, 161–163).

Table S3. The number of fish composites within each of our habitat and diet classifications.

**Diet**	**Habitat**				
	Benthic	Benthopelagic	Midwater	Pelagic	**Total**
Herbivore	-	20	6	-	**26**
Primary Carnivore	-	67	56	89	**212**
Secondary Carnivore	83	357	143	68	**651**
Tertiary Carnivore	47	134	1	3	**185**
**Total**	**130**	**578**	**206**	**160**	**1074**

Table S4. Model selection using ΔAIC (Akaike information criteria) and 5-fold-cross-validation (CV) for alternative models predicting [DDX_{sed}]. To conduct cross-validation we split the data into training sets (80% of the data) and testing sets (20% of the data) using unstratified random sampling. We then re-ran each spatiotemporal model using only the training data. We simulated DDX concentrations for the testing data by simulating 500 draws from the joint precision matrix, taking the median estimated value, and calculating the mean R^2 and mean squared error (MSE) as a measure of out-of-sample predictive ability. Metrics were calculated on log(x+ 1) transformed data, where x is the sediment DDX concentration. Simulating data from the joint precision matrix, rather than using the expected value for a point, more accurately captured the expected distribution of zeroes and allowed us to report a single value for both models.

Fixed Effects	Random Effects	ΔAIC	CV R^2	CV MSE
	Spatial	624.05	0.58	1.19
Year	Spatial	291.99	0.63	1.06
Depth	Spatial	520.34	0.61	1.12
Year, Depth	Spatial	171.49	0.66	0.99
	Spatiotemporal AR(1)	178.22	0.70	0.85
Year	Spatiotemporal AR(1)	141.63	0.71	0.81
Depth	Spatiotemporal AR(1)	27.39	0.72	0.79
**Year, Depth**	**Spatiotemporal AR(1)**	**0.00**	**0.74**	**0.76**

Table S5. Estimated coefficients and 90% Confidence Intervals from the beset-fit model of sediment DDX concentrations.

	**Maximum Likelihood Estimate**		**Confidence Interval**	
**Model type**	Encounter	Concentration	Encounter	Concentration
**Year effects**				
2003	-0.88	1.28	(-3.38, 1.61)	(0.49, 2.08)
2008	3.15	1.94	(0.58, 5.71)	(1.21, 2.67)
2013	3.76	1.18	(0.92, 6.59)	(0.43, 1.93)
2018	5.57	1.62	(2.52, 8.62)	(0.88, 2.36)
**Spatial Parameters**				
Matern range	70.3	36.3	(42.0, 118.0)	(29.4, 44.9)
Marginal spatial standard deviation	18.6	4.80	(8.86, 38.9)	(3.51, 6.57)
Spatiotemporal AR1 correlation	0.86	0.80	(0.61, 0.96)	(0.70, 0.87)
**Gamma Dispersion**	-	1.88	-	(1.67, 2.11)

Table S6. Fish Advisory Tissue Levels (ATLs) developed by the California EPA's Office of Environmental Health Hazard Assessment (OEHHA) for DDX based on carcinogenic or non-carcinogenic risk using an 8-ounce serving size per week prior to cooking (164). Values are in ng g^{-1} wet weight. Fish Contaminant Goals (FCGs) are estimates of contaminant levels in fish that pose no significant health risk to individuals consuming sport fish at a standard consumption rate of eight ounces per week (32 g/day), prior to cooking, over a lifetime and can provide a starting point for OEHHA to assist other agencies that wish to develop fish tissue-based criteria with a goal toward pollution mitigation or elimination.

	**FCG**	**ATLs for the number of 8-oz servings per week (ng g^{-1} wet weight)**							
		7	6	5	4	3	2	1	Do not consume
Threshold	≤ 21	≤ 220	220-260	260-310	310-390	390-520	520-1000	1000-2100	≥ 2100
Number of composites within each category	582	926	8	11	13	10	33	37	36
Percent of composites within each category	54.2	86.2	0.7	1.0	1.2	0.9	3.1	3.4	3.4

Supporting Information References

1. F. Hartig, DHARMa: Residual Diagnostics for Hierarchical (Multi-Level / Mixed) Regression Models. (2022). Deposited 2022.

2. F. Rodríguez-Sánchez, DHARMa.helpers: Helper functions to check models not (yet) directly supported by DHARMa. (2023). Deposited 2023.

3. C. Liao, A. R. Taylor, W. F. Kenney, D. Schlenk, J. Gan, Historical record and fluxes of DDTs at the Palos Verdes Shelf Superfund site, California. *Sci. Total Environ.* **581–582**, 697–704 (2017).

4. J. T. Schmidt, *et al.*, Disentangling the History of Deep Ocean Disposal for DDT and Other Industrial Waste Off Southern California. *Environ. Sci. Technol.* **58**, 4346–4356 (2024).

5. C. Beegan, S. M. Bay, Transitioning sediment quality assessment into regulations: Challenges and solutions in implementing California's sediment quality objectives. *Integr. Environ. Assess. Manag.* **8**, 586–588 (2012).

6. D. M. Walters, *et al.*, Trophic Magnification of Organic Chemicals: A Global Synthesis. *Environ. Sci. Technol.* **50**, 4650–4658 (2016).

7. W. M. Meylan, *et al.*, Improved method for estimating bioconcentration/bioaccumulation factor from octanol/water partition coefficient. *Environ. Toxicol. Chem.* **18**, 664–672 (1999).

8. A. R. Taylor, J. Wang, C. Liao, D. Schlenk, J. Gan, Effect of aging on bioaccessibility of DDTs and PCBs in marine sediment. *Environ. Pollut.* **245**, 582–589 (2019).

9. J. Wang, A. Taylor, D. Schlenk, J. Gan, Application and validation of isotope dilution method (IDM) for predicting bioavailability of hydrophobic organic contaminants in soil. *Environ. Pollut.* **236**, 871–877 (2018).

10. Southern California Bight Regional Monitoring Pogram. *Bight Data Portal*. Available at: https://bight.sccwrp.org/ [Accessed 2 July 2024].

11. J. A. Davis, *et al.*, "Contaminants in Fish from the California Coast, 2009: Summary Report on Year One of a Two-Year Screening Survey" (California State Water Resources Control Board, 2011).

12. K. McLaughlin, *et al.*, Regional assessment of contaminant bioaccumulation in sport fish tissue in the Southern California Bight, USA. *Mar. Pollut. Bull.* **172**, 112798 (2021).

13. M. Gassel, R. K. Brodberg, S. Roberts, The Coastal Fish Contamination Program: Monitoring of Coastal Water Quality and Chemical Contamination in Fish and Shellfish in California in *California and the World Ocean '02*, (American Society of Civil Engineers, 2005), pp. 977–990.

14. E. Jarvis, K. Schiff, L. Sabin, M. J. Allen, Chlorinated hydrocarbons in pelagic forage fishes and squid of the Southern California Bight. *Environ. Toxicol. Chem.* **26**, 2290 (2007).

15. NOAA, “2002-2004 Southern California Coastal Marine Fish Survey” (National Oceanic Atmospheric and Administration, 2007).

16. Los Angeles County Sanitation Districts Ocean Monitoring Program, “A.K. Warren Water Resource Facility Biennial Receiving Water Monitoring Report 2022-2023” (2024).

17. City of San Diego Ocean Monitoring Program, “Biennial recieving waters monitoring and assessment report for the Point Loma and South Bay ocean outfalls, 2022-2023” (2024).

18. B. B. McCain, *et al.*, “Life histories, geographical distributions, and habitat associations of Pacific coast groundfish species\” in *Pacific Coast Groundfish Fishery Management Plan for the California, Oregon, and Washington Groundfish Fishery*, (Pacific Fishery Management Council, 2019).

19. M. H. Horn, L. A. Ferry-Graham, “Feeding Mechanisms and Trophic Interactions” in *The Ecology of Marine Fishes*, California and Adjacent Waters., 1st Ed., M. H. Horn, L. G. Allen, D. J. Pondella, Eds. (University of California Press, 2006), pp. 387–410.

20. D. A. Roberts, E. E. DeMartini, K. M. Plummer, The feeding habits of juvenile-small adult barred sand bass (Paralabrax nebulifer) in nearshore waters off northern San Diego County. *CalCOFI Rep.* **25** (1984).

21. T. D. Johnson, *et al.*, Fish Production and Habitat Utilization on a Southern California Artificial Reef. *Bull. Mar. Sci.* **55**, 709–723 (1994).

22. M. J. Allen, *Functional Structure of Soft-bottom Fish Communities of the Southern California Shelf* (University of California, San Diego, 1982).

23. M. J. Allen, “Continental Shelf and Upper Slope” in *The Ecology of Marine Fishes*, California and Adjacent Waters., 1st Ed., L. G. Allen, D. J. Pondella, M. H. Horn, Eds. (University of California Press, 2006), pp. 167–202.

24. J. S. Stephens, R. J. Larson, D. J. Pondella, “Rocky Reefs and Kelp Beds” in *The Ecology of Marine Fishes*, California and Adjacent Waters., 1st Ed., D. J. Pondella, L. G. Allen, M. H. Horn, Eds. (University of California Press, 2006), pp. 227–252.

25. A. W. Ebeling, R. J. Larson, W. S. Alevizon, Habitat Groups and Island-Mainland Distribution of Kelp-bed Fishes off Santa Barbara, CA in (1980).

26. T. J. Mason, C. G. Lowe, Home range, habitat use, and site fidelity of barred sand bass within a southern California marine protected area. *Fish. Res.* **106**, 93–101 (2010).

27. L. G. Allen, D. J. Pondella, “Ecological Classification” in *The Ecology of Marine Fishes*, California and Adjacent Waters., 1st Ed., L. G. Allen, D. J. Pondella, M. H. Horn, Eds. (University of California Press, 2006), pp. 81–114.

28. California Department of Fish and Wildlife, “Barred Surfperch, Amphistichus argenteus, and Redtail Surfperch, Amphistichus rhodoterus” (2019).

29. J. R. Madden, “Temporal variation and ontogenetic shifts in the diet of a surf zone fish, the barred surfperch, Amphistichus argenteus” (2023).

30. J. G. Carlisle, J. W. Schott, N. J. Abramson, “The Barred Surfperch (Amphistichus argenteus Agassiz) in Southern California” (1960).

31. K. J. Nielsen, *et al.*, “Baseline Characterization of Sandy Beach Ecosystems along the North Coast of California” (2017).

32. E. DeMartini, A correlative study of the ecology and comparative feeding mechanism morphology of the Embiotocidae (surf-fishes) as evidence of the family’s adaptive radiation into available ecological niches. *Wasmann J Biol* **27**, 177–247 (1969).

33. C. H. Turner, E. E. Ebert, R. R. Given, Man-Made Reef Ecology. *Fish Bull.* **146** (1969).

34. J. P. Ellison, C. Terry, J. S. Stephens, Food resource utilization among five species of embiotocids and King Harbor, California, with preliminary estimates of caloric intake. *Mar. Biol.* **58**, 914–920 (1979).

35. J. J. Bizzarro, *et al.*, A multi-predator trophic database for the California Current Large Marine Ecosystem. *Sci. Data* **10**, 496 (2023).

36. M. A. Hixon, Competitive Interactions between California Reef Fishes of the Genus Embiotoca. *Ecology* **61**, 918–931 (1980).

37. D. W. Gotshall, J. G. Smith, A. Holbert, “Food of the blue rockfish Sebastes mystinus” (California Department of Fish and Game, 1965).

38. J. L. Hart, *Pacific fishes of Canada* (Fisheries Research Board of Canada, 1973).

39. M. S. Love, W. Ebeling, Food and habitat of three switch-feeding fishes in the kelp forests off Santa Barbara. *Fish. Bull.* **76**, 257–271 (1978).

40. J. S. Macgregor, “Growth of the blue rockfish (Sebastes mystinus)” (1983).

41. E. S. Hobson, J. R. Chess, D. F. Howard, Interannual variation in predation on five-year Sebastes spp. by three northern California predators. *Fish. Bull.* **99**, 292–302 (2000).

42. C. A. Sullivan, “Grouping of fishing locations using similarities in species compositions for the Monterey Bay area commercial passenger fishing vessel fishery, 1987-1992” (1995).

43. R. N. Lea, “Rockfishes: Overview” in *California’s Living Marine Resources and Their Utilization*, W.S. Leet, C. M. Dewees, C. W. Haugen, Eds. (California Sea Grant College Program, 1992), pp. 114–117.

44. M. S. Love, M. H. Carr, L. J. Haldorson, The ecology of substrate-associated juveniles of the genus Sebastes. *Environ. Biol. Fishes* **30**, 225–243 (1991).

45. R. N. Lea, R. D. McAllister, D. A. Ven Tresca, “Biological aspects of nearshore rockfishes of the genus Sebastes from central California” (California Department of Fish and Game, 1999).

46. D. Stein, T. J. Hassler, “Species profiles: Life histories and environmental requirements of coastal fishes and invertebrates(Pacific southwest): Brown rockfish, copper rockfish, and black rockfish.” (US Fish and Wildlife Service, 1989).

47. M. S. Love, *Probably More Than You Want to Know About the Fishes of the Pacific Coast* (Really Big Press, 1996).

48. J. G. Carlisle Jr., C. H. Turner, E. E. Ebert, “Artificial habitat in the marine environment” (California Department of Fish and Game, 1964).

49. H. M. Feder, C. H. Turner, C. Limbaugh, “Observations on fishes associated with kelp beds in southern California” (California Department of Fish and Game, 1974).

50. P. M. Washington, R. Gowan, D. H. Ito, “A biological report on eight species of rockfish (Sebastes spp.) from Puget Sound, Washington.” (US Department of Commerce, NOAA/NMFS, 1978).

51. R. M. Buckley, G. J. Hueckel, Biological processes and ecological development on an artificial reef in Puget Sound, Washington. *Bull. Mar. Sci.* **37**, 50–69 (1985).

52. S. J. Holbrook, R. J. Schmitt, J. S. Stephens, Changes in an assemblage of temperate reef fishes associated with a climate shift. *Ecol. Appl.* **7**, 1299–1310 (1997).

53. K. R. Matthews, A Comparative Study of Habitat Use by Young-of-the-year, Subadult, and Adult Rockfishes on Four Habitat Types in Central Puget Sound. *Fish. Bull.* **88**, 223–239 (1990).

54. K. R. Matthews, An experimental study of the habitat preferences and movement patterns of copper, quillback, and brown rockfishes (Sebastes spp.). *Environ. Biol. Fishes* **29**, 161–178 (1990).

55. J. E. Mason, Species trends in sport fisheries, Monterey Bay, Calif., 1959-86. *Mar. Fish. Rev.* **57**, 1+ (1995).

56. M. Espinoza, T. M. Clarke, F. Villalobos-Rojas, I. S. Wehrtmann, Ontogenetic dietary shifts and feeding ecology of the rasptail skate *Raja velezi* and the brown smoothhound shark *Mustelus henlei* along the Pacific coast of Costa Rica, Central America. *J. Fish Biol.* **81**, 1578–1595 (2012).

57. D. F. Amariles, A. F. Navia, A. Giraldo, Food resource partitioning of the Mustelus lunulatus and Mustelus henlei (Elasmobranchii: Carcharhiniformes). *Environ. Biol. Fishes* **100**, 717–732 (2017).

58. L. M. Pantoja-Echevarría, *et al.*, Stable isotopic inferences on trophic ecology and habitat use of brown smooth-hound Mustelus henlei in the west coast of Baja California Sur, Mexico. *Reg. Stud. Mar. Sci.* **40**, 101520 (2020).

59. K. G. Yudin, G. M. Cailliet, Age and Growth of the Gray Smoothhound, Mustelus californicus, and the Brown Smoothhound, M. henlei, Sharks from Central California. *Copeia* **1990**, 191 (1990).

60. California Department of Fish and Wildlife, “California Corbina, Menticirrhus undulatus” (2022).

61. T. Skogsberg, The Fishes of the Family Sciaenidae (Croakers) of California. *Fish Bull.* **54**, 62 (1939).

62. D. C. Joseph, Fish Bulletin No. 119. Growth Characteristics of Two Southern California Surffishes, the California Corbina and Spotfin Croaker, Family Sciaenidae. *Fish Bull.* **119**, 1–54 (1962).

63. J. O'Brien, C. Valle, Food habits of California Corbina in Southern California. *Calif. Fish Game* **86**, 136–148 (2000).

64. S. P. Wertz, M. L. Domeier, Relative importance of prey items to California Halibut. *Calif. Fish Game* **83**, 21–29 (1997).

65. California Department of Fish and Wildlife, "California halibut, Paralichthys californicus" (2022).

66. Southern California Coastal Water Research Project, "Dietary Guild and Target Species Development for SQO Indirect Effects Assessment" (2010).

67. J. E. Fitch, R. J. Lavenberg, *Marine food and game fishes of California* (University of California Press, 1971).

68. P. Taylor, *The venom and ecology of the California scorpionfish, Scorpaena guttata Girard* (University of California, San Diego, 1963).

69. E. S. Hobson, W. N. McFarland, J. R. Chess, Crepuscular and nocturnal activities of californian nearshore fishes, with consideration of their scotopic visual pigments and the photic environment. *Fish Bull.* **79**, 1–30 (1981).

70. M. J. Paddack, J. A. Estes, Kelp forest fish populations in marine reserves and adjacent exploited areas of central California. *Ecol. Appl.* **10**, 855–870 (2000).

71. P. Quast, Observations on the food of the kelp-bed fishes. *Calif. Dep. Fish Game Fish Bull.* **139**, 109–142 (1968).

72. G. W. Boehlert, M. M. Yoklavich, D. B. Chelton, Time series of growth in the genus Sebastes from the northeast Pacific ocean. *Fish Bull.* **87**, 791–806 (1989).

73. M. J. Allen, G. B. Smith, "Atlas and zoogeography of common fishes in the Bering Sea and northeastern Pacific" (U.S. Dept. of Commerce, 1988).

74. J. W. Orr 1958-, M. A. (Michael A. Brown 1938-, D. C. Baker, Guide to rockfishes (Scorpaenidae) of the genera Sebastes, Sebastolobus, and Adelosebastes of the northeast Pacific Ocean. (1998).

75. C. Reilly, T. Echeverria, S. Ralston, Interannual variation and overlap in the diets of pelagic juvenile rockfish (Genus: SebastesJ off central California. *Fish Bull.* **90** (1992).

76. M. Love, P. Morris, M. McCrae, R. Collins, Life History Aspects of 19 Rockfish Species (Scorpaenidae: Sebastes) from the Southern California Bight. *NOAA Tech Rep NMFS* **87** (1990).

77. E. S. Konno, P. Wolf, "Pacific mackerel" in *California's Living Marine Resources: A Status Report*, W. S. Leet, C. M. Dewees, R. Klingbeil, E. J. Larson, Eds. (California Department of Fish and Game, 2001), pp. 306–308.

78. K. F. Mais, "Pelagic Fish Surveys In The California Current" (California Department of Fish and Game, 1974).

79. E. Prince, D. Gotshall, Food of the copper rockfish, Sebastes caurinus, Richardson, associated with an artificial reef in South Humboldt Bay, California. *Calif. Fish Game* **62**, 275–285 (1976).

80. K. R. Matthews, A telemetric study of the home ranges and homing routes of copper and quillback rockfishes on shallow rocky reefs. *Can. J. Zool.* **68**, 2243–2250 (1990).

81. R. N. Lea, "Copper rockfish" in *California's Living Marine Resources and Their Utilization*, W.S. Leet, C. M. Dewees, C. W. Haugen, Eds. (California Sea Grant College Program, 2001), pp. 173–174.

82. M. S. Love, M. Yoklavich, L. K. Thorsteinson, *The Rockfishes of the Northeast Pacific* (University of California Press, 2002).

83. D. J. Miller, J. J. Geibel, "Summary of blue rockfish and lingcod life histories; a reef ecology study; and giant kelp, Macrocystis pyrifera, experiments in Monterey Bay, California" (California Department of Fish and Game, 1973).

84. A. R. Hartmann, Movement of scorpionfishes (Scorpaenidae: Sebastes and Scorpaena) in the Southern California Bight. *Calif. Dep. Fish Game* **73**, 68–79 (1987).

85. California Department of Fish and Wildlife, "Diamond Turbot, Hypsopsetta guttulata" (2023).

86. D. J. Miller, R. N. Lea, Guide To The Coastal Marine Fishes of California. *Calif. Dep. Fish Game Fish Bull.* **157**, 249 (1972).

87. J. W. Orr, M. A. Brown, D. C. Baker, Guide to Rockfishes (Scorpaenidae) of the Genera Sebastes, Sebastolobus, and Adelosebastes of the Northeast Pacific Ocean, Second Edition in (1998).

88. California Dept. Fish and Game, Marine Life Protection Act: Species likely to benefit from the establishment of marine protected areas in California. *Web Site Manag. Paul Reilly* (2003).

89. A. W. Ebeling, R. N. Bray, Day versus night activity of reef fishes in a kelp foreset off Santa Barbara, California. *Calif. Dep. Fish Game Fish Bull.* **74**, 703–717 (1976).

90. R. J. Larson, Territorial behavior of the black and yellow rockfish and gopher rockfish (Scorpaenidae, Sebastes). *Mar. Biol.* **58**, 111–122 (1980).

91. R. J. Larson, Competition, Habitat Selection, and the Bathymetric Segregation of Two Rockfish (Sebastes) Species. *Ecol. Monogr.* **50**, 221–239 (1980).

92. M. H. Carr, Habitat selection and recruitment of an assemblage of temperate zone reef fishes. *J. Exp. Mar. Biol. Ecol.* **146**, 113–137 (1991).

93. K. L. Weinberg, The 2001 Pacific west coast bottom trawl survey of groundfish resources : estimates of distribution, abundance, and length and age composition. (2002).

94. W. N. Eschmeyer, E. S. Herald, *A Field Guide to Pacific Coast Fishes: North America* (Houghton Mifflin Harcourt, 1983).

95. California Department of Fish and Wildlife, "Halfmoon, Medialuna californiensis" (2020).

96. M. E. Díaz-Díaz, M. G. Hammann, Trophic Relations Among Fishes Associated To A Kelp Forest, Macrocystis Pyrifera, In Bahia De Todos Santos, Baja California, Mexico. *Cienc. Mar.* **13**, 81–96 (1987).

97. S. Holbrook, M. Carr, R. Schmitt, J. Coyer, Effect of Giant Kelp on Local Abundance of Reef Fishes: The Importance of Ontogenetic Resource Requirements. *Bull. Mar. Sci.* **47**, 104–114 (1990).

98. L. E. Hallacher, D. A. Roberts, Differential utilization of space and food by the inshore rockfishes (Scorpaenidae: Sebastes) of Carmel Bay, California. *Environ. Biol. Fishes* **12**, 91–110 (1985).

99. L. Talent, Food habits of the leopard shark, Triakis semifasciata, in Elkhorn Slough, Monterey Bay, California. *Calif. Fish Game* **62**, 286–298 (1976).

100. D. A. Ebert, T. B. Ebert, Reproduction, diet and habitat use of leopard sharks, Triakis semifasciata (Girard), in Humboldt Bay, California, USA. *Mar. Freshw. Res.* **56**, 1089 (2005).

101. L. J. V. Compagno, "Part 2: Carcharhiniformes" in *Sharks of the World: An Annotated and Illustrated Catalogue of Shark Species Known to Date*, FAO fisheries synopsis., (United Nations Development Programme, 1984), pp. 251–655.

102. R. Russo, Observations on the food habits of leopard sharks (Triakis semifasciata) and brown smoothhounds (Mustelus henlei). *Calif Fish Game* **61**, 95–103 (1975).

103. A. Ferguson, G. M. Cailliet, *Sharks and rays of the Pacific Coast* (Monterey Bay Aquarium, 1990).

104. R. L. (Robert L. Emmett, Distribution and abundance of fishes and invertebrates in West Coast estuaries. Volume II, Species life history summaries. (1991).

105. S. E. Smith, "Leopard Shark" in *California's Living Marine Resources and Their Utilization*, W.S. Leet, C. M. Dewees, C. W. Haugen, Eds. (California Sea Grant College Program, 2001), pp. 173–174.

106. S. Smith, N. Abramson, Leopard Shark Triakis semifasciata Distribution, Mortality Rate, Yield, and Stock Replenishment Estimates Based on a Tagging Study in San Francisco Bay. *Fish. Bull. Natl. Ocean. Atmospheric Adm.* **88**, 371–381 (1990).

107. M. Yaremko, "Market squid" in *California's Living Marine Resources: A Status Report*, W. S. Leet, C. M. Dewees, R. Klingbeil, E. J. Larson, Eds. (California Department of Fish and Game, 2001), pp. 295–298.

108. L. D. Zeidberg, W. M. Hamner, N. P. Nezlin, A. Henry, "The fishery for California market squid (Loligo opalescens) (Cephalopoda: Myopsida), from 1981 through 2003" (2004).

109. D. R. Bergen, L. D. Jacobson, "Northern anchovy" in *California's Living Marine Resources: A Status Report*, W. S. Leet, C. M. Dewees, R. Klingbeil, E. J. Larson, Eds. (California Department of Fish and Game, 2001), pp. 303–305.

110. California Department of Fish and Wildlife, "Ocean whitefish, Caulolatilus princeps" (2019).

111. L. Bellquist, C. Lowe, J. Caselle, Fine-scale movement patterns, site fidelity, and habitat selection of ocean whitefish (Caulolatilus princeps). *Fish. Res.* **91**, 325–335 (2008).

112. M. Love, Isolation of olive rockfish, Sebastes serranoides, populations off southern California. *Fish Bull* **77**, 975–983 (1980).

113. J. C. Quast, Fish fauna of the rocky inshore zone. *Calif. Dep. Fish Game Fish Bull.* **139**, 109–142 (1968).

114. J. L. Bodkin, Effects of kelp forest removal on associated fish assemblages in central California. *J. Exp. Mar. Biol. Ecol.* **117**, 227–238 (1988).

115. D. L. Watters, "Olive rockfish" in *California's Living Marine Resources and Their Utilization*, W.S. Leet, C. M. Dewees, C. W. Haugen, Eds. (California Sea Grant College Program, 1992), pp. 173–174.

116. M. D. Behrens, K. D. Lafferty, Geographic Variation in the Diet of Opaleye (Girella nigricans) with Respect to Temperature and Habitat. *PLoS ONE* **7**, e45901 (2012).

117. California Department of Fish and Wildlife, "Pacific Barracuda, Sphyraena argentea" (2022).

118. E. DeMartini, L. Allen, R. Fountain, D. Roberts, Diel and depth variation in the sex-specific abundance, size composition and food habits of queenfish Seriphus politus Sciaenidae. U.S. *Natl. Mar. Fish. Serv. Fish. Bull.* **83** (1985).

119. E. S. Hobson, J. R. Chess, Relationships among fishes and their prey in a nearshore sand community off southern California. *Environ. Biol. Fishes* **17**, 201–226 (1986).

120. R. J. Rosenthal, V. Moran-O'Connell, M. C. Murphy, Feeding ecology of ten species of rockfishes (Scorpaenidae) from the Gulf of Alaska. *Calif. Dep. Fish Game Fish Bull.* **74**, 16–36 (1988).

121. G. Hueckel, L. Stayton, Fish Foraging on an Artificial Reef in Puget Sound, Washington. *Mar. Fish. Rev.* **44** (1982).

122. D. J. Murie, Comparative feeding ecology of two sympatric rockfish congeners, Sebastes caurinus (copper rockfish) and S. maliger (quillback rockfish). *Mar. Biol.* **124**, 341–353 (1995).

123. K. R. Matthews, *Habitat use and movement patterns of copper, quillback, and brown rockfishes in Puget Sound, Washington* (University of Washington, 1988).

124. D. Gascon, R. A. Miller, Colonization by nearshore fish on small artificial reefs in Barkley Sound, British Columbia. *Can. J. Zool.* **59**, 1635–1646 (1981).

125. J. B. Phillips, A review of the rockfishes of California (Family Scorpaenidae). *Calif. Dep. Fish Game Fish. Bull.* **104**, 158 (1957).

126. D. L. Alverson, *A study of demersal fishes and fisheries of the northeastern Pacific Ocean* (1967).

127. S. L. Richardson, W. A. Laroche, Development and occurrence of larvae and juveniles of the rockfishes Sebastes crameri, Sebastes pinniger, and Sebastes helvomaculatus (Family Scorpaenidae) off Oregon. *Fish. Bull.* **77**, 1–46 (1979).

128. D. B. Odenweller, Aspects of the biology of the shiner perch in a Southern California estuary. *Trans. West. Sect. Wildl. Soc.* **11**, 93–101 (1975).

129. R. H. Chamberlain, R. A. Barnhart, Early Use by Fish of a Mitigation Salt Marsh, Humboldt Bay, California. *Estuaries* **16**, 769 (1993).

130. S. DeLeón, "Report on the 1980-1995 Fish, Shrimp, and Crab Sampling in the San Francisco Estuary, California" in *Embiotocidae*, Technical Report., J. J. Orsi, Ed. (Interagency Ecological Program for the Sacramento-San Joaquin Estuary, 1999), pp. 1–503.

131. M. S. Love, Certainly More Than You Want to Know About The Fishes of The Pacific Coast: A Postmodern Experience. *Really Big Press* 672 (2011). https://doi.org/10.1643/OT-12-002.1.

132. K. Bannister, *The Book of the Shark* (Apple Press, 1989).

133. D. A. Ebert, *Sharks, Rays, and Chimaeras of California* (University of California Press, 2003).

134. B. C. Jones, G. H. Geen, Food and Feeding of Spiny Dogfish (*Squalus acanthias*) in British Columbia Waters. *J. Fish. Res. Board Can.* **34**, 2056–2066 (1977).

135. J. Castro, *The Sharks of North American Waters* (Texas A&M University Press, 1983).

136. L. G. Allen, T. E. Hovey, M. S. Love, J. T. Smith, *Calif. Coop. Ocean. Fish. Investig. CalCOFI Rep.* **36**, 193–203 (1995).

137. M. Mendoza-Carranza, J. A. Rosales-Casian, The feeding habits of spotted sand bass (Paralabrax maculatofasciatus) in Punta Banda estuary, Ensenada, Baja California, Mexico. *Calif. Coop. Ocean. Fish. Investig. CalCOFI Rep.* **41**, 194–200 (2000).

138. L. Allen, A. Findlay, C. Phalen, Structure and Standing Stock of the Fish Assemblages of San Diego Bay, California from 1994 to 1999. *Bull. South. Calif. Acad. Sci.* **101**, 49–85 (2002).

139. M. Love, P. Morris, M. McCrae, R. Collins, Life History Aspects of 19 Rockfish Species (Scorpaenidae: Sebastes) from the Southern California Bight. *NOAA Tech Rep NMFS* **87** (1990).

140. S. Blanco, S. Romo, M.-J. Villena, S. Martínez, Fish communities and food web interactions in some shallow Mediterranean lakes. *Hydrobiologia* **506–509**, 473–480 (2003).

141. R. D. S. Wells, The food of the grey mullet (*Mugil cephalus* L.) in Lake Waahi and the Waikato River at Huntly. *N. Z. J. Mar. Freshw. Res.* **18**, 13–19 (1984).

142. L. G. Allen, *Structure and productivity of the littoral fish assemblage of Upper Newport Bay, California* (University of Southern California Los Angeles, 1980).

143. T. Visintainer, S. Bollens, C. Simenstad, Community composition and diet of fishes as a function of tidal channel geomorphology. *Mar. Ecol. Prog. Ser.* **321**, 227–243 (2006).

144. B. K. Greenfield, A. Jahn, Mercury in San Francisco Bay forage fish. *Environ. Pollut.* **158**, 2716–2724 (2010).

145. J. R. Hyde, R. D. Vetter, Population genetic structure in the redefined vermilion rockfish (Sebastes miniatus) indicates limited larval dispersal and reveals natural management units. *Can. J. Fish. Aquat. Sci.* **66**, 1569–1581 (2009).

146. G. M. Cailliet, *et al.*, "Biological characteristics of nearshore fishes of California: A review of existing knowledge and proposed additional studies" (Moss Landing Marine Laboratories, 2000).

147. E. S. Hobson, J. R. Chess, Trophic interactions among fishes and zooplakters nearshore at Santa Catalina island, California. *Fish. Bull.* **73** (1976).

148. R. R. Ware, "The food habits of the White croaker Genyonemus lineatus and an infaunal analysis near areas of waste discharge in outer Los Angeles Harbor," California State University, Long Beach. (1979).

149. F. A. P. C. Gobas, J. A. Arnot, Food web bioaccumulation model for polychlorinated biphenyls in San Francisco Bay, California, USA. *Environ. Toxicol. Chem.* n/a-n/a (2010). https://doi.org/10.1002/etc.164.

150. B. W. Wolfe, C. G. Lowe, Movement patterns, habitat use and site fidelity of the white croaker (Genyonemus lineatus) in the Palos Verdes Superfund Site, Los Angeles, California. *Mar. Environ. Res.* **109**, 69–80 (2015).

151. R. N. Bray, A. W. Ebeling, Food, activity, and habitat of three "picker type" microcarnivorous fishes in the kelp forests of Santa Barbara, California in (1975).

152. A. Ebling, R. Bray, Day versus night activity of reef fishes in a kelp forest off Santa Barbara, California. *Fish. Bull.* **74**, 703–717 (1976).

153. D. J. Pondella, *et al.*, Demographic Parameters of Yellowfin Croaker, Umbrina roncador (Perciformes: Sciaenidae), from the Southern California Bight [1]. *Pac. Sci.* **62**, 555–568 (2008).

154. J. A. Chiu, J. J. Bizzarro, R. M. Starr, Trophic ecology of yellowtail rockfish (Sebastes flavidus) during a marine heat wave off central California, USA. *PLOS ONE* **16**, e0251499 (2021).

155. J. B. Phillips, Life History Studies on Ten Species of Rockfish (Genus Sebastodes). *Calif. Dep. Fish Game Fish Bull.* **126**, 70 (1964).

156. J. Tagart, "Population dynamics of yellowtail rockfish (Sebastes flavidus) stocks in the northern California to southwest Vancouver Island region /," University of Washington. (1991).

157. H. R. Carlson, R. E. Haight, Evidence for a Home Site and Homing of Adult Yellowtail Rockfish, *Sebastes flavidus*. *J. Fish. Res. Board Can.* **29**, 1011–1014 (1972).

158. M. E. Fraidenburg, First Estimates of Natural Mortality for Yellowtail Rockfish. *Trans. Am. Fish. Soc.* **110**, 551–553 (1981).

159. K. L. Weinberg, Rockfish assemblages of the middle shelf and upper slope off Oregon and Washington | Scientific Publications Office. *Fish Bull.* **92**, 620–632 (1994).

160. W. A. Laroche, S. L. Richardson, Development of larvae and juveniles of the rockfishes Sebastes entomelas and S.zacentrus(Family Scorpaenidae) and occurrence off Oregon, with notes on head spines of S. mystinus, S. flavidus, and S. melanops. *Fish Bull.* **92**, 231–256 (1981).

161. R. D. Stanley, B. M. Leaman, L. Haldorson, V. M. O'Connell, Movements of tagged adult yellowtail rockfish, Sebastes flavidus, off the west coast of North America. *Fish Bull.* **92**, 655–663 (1994).

162. H. R. Carlson, Restricted Year-Class Structure and Recruitment Lag within a Discrete School of Yellowtail Rockfish. *Trans. Am. Fish. Soc.* **115**, 490–492 (1986).

163. W. G. Pearcy, Movements of acoustically-tagged yellowtail rockfish Sebastes flavidus on Heceta Bank, Oregon. *Fish Bull.* **90**, 726–735 (1992).

164. S. Klasing, R. Brodberg, "Development of Fish Contaminant Goals and Advisory Tissue Levels for Common Contaminants in California Sport Fish: Chlordane, DDTs, Dieldrin, Methylmercury, PCBs, Selenium, and Toxaphene" (California Office of Health Hazards Assessment, 2008).
